# Tuning trityl radical content in multivariate metal–organic radical frameworks

**DOI:** 10.1039/d6qm00770h

**Published:** 2026-09-30

**Authors:** Tianshu Zhang, Gonçalo Valente, Pavel Zelenovskii, Ricardo F. Mendes, Manuel Souto

**Affiliations:** a CiQUS, Centro Singular de Investigación en Química Biolóxica e Materiais Moleculares, Departamento de Química-Física, Universidade de Santiago de Compostela 15782 Santiago de Compostela Spain ricardo.faria@usc.es manuel.souto.salom@usc.es; b CICECO-Aveiro Institute of Materials, University of Aveiro 3810-393 Aveiro Portugal; c Oportunius, Galician Innovation Agency (GAIN) 15702 Santiago de Compostela Spain

## Abstract

Persistent organic radicals are attractive building blocks for constructing functional metal–organic frameworks owing to their unique electronic, magnetic, and optical properties. However, tuning the ratio of open- and closed-shell organic linkers within a single metal–organic radical framework (MORF) has remained unexplored. Herein, we report the synthesis of multivariate MORFs incorporating perchlorotriphenylmethyl tricarboxylate (PTMTC) linkers in both their radical (PTMTC^R^) and non-radical (PTMTC^NR^) forms within the same crystalline structure. By simply varying the ratio of the starting PTMTC^R^ and PTMTC^NR^ ligands, we obtained three different multivariate MORFs (MORF-40, MORF-60, and MORF-80) with radical contents of *ca*. 40, 60, and 80%, respectively, while preserving the same framework architecture. Single-crystal X-ray diffraction directly reveals the statistical co-occupancy of PTMTC^R^ and PTMTC^NR^ linkers at the same crystallographic site in MORF-40, providing a rare example of a crystalline framework containing both open- and closed-shell organic building blocks. Complementary Raman spectroscopy, electron paramagnetic resonance, fluorescence microscopy, and Kelvin probe force microscopy confirm the systematic variation in radical content across the series. This ability to tune the radical content without altering the underlying framework architecture provides a versatile strategy for modulating the electronic, optical, and magnetic properties of multivariate metal–organic radical frameworks.

## Abstract 2

Multivariate metal–organic radical frameworks with tunable open-shell linker contents are reported. Varying radical/non-radical PTMTC ratios yields isostructural MORFs, enabling control of their electronic, optical, and magnetic properties.

## Graphical abstract

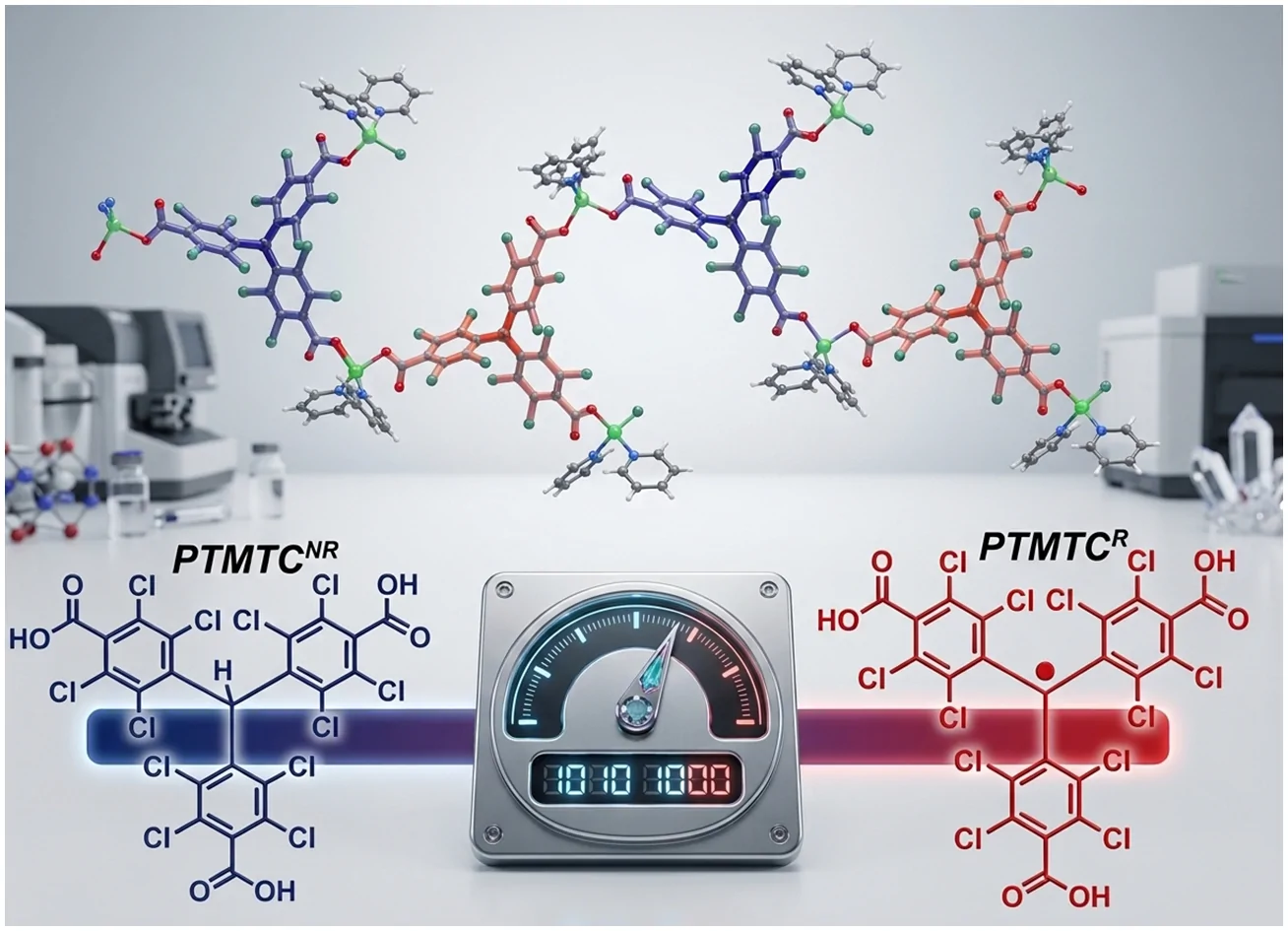

## Introduction

Persistent organic radicals are open-shell molecules with unique electronic, optical, and magnetic properties that have attracted considerable interest for applications in electronics, spintronics, and energy storage.^1–3^ Among persistent organic radicals, polychlorinated trityl radicals such as perchlorotriphenylmethyl (PTM) radical derivatives have emerged as versatile open-shell molecular building blocks for advanced optoelectronic and spin-based technologies, including organic light-emitting diodes, spintronic, and molecular memory devices.^4–7^

Stable neutral π-radicals represent attractive building blocks for the construction of metal–organic frameworks (MOFs) and coordination polymers, enabling the integration of their intrinsic electronic, optical, and magnetic properties into extended crystalline architectures.^8,9^ Although the incorporation of neutral organic radical linkers into MOFs remains relatively unexplored, PTM radicals have been successfully employed to construct metal–organic radical frameworks (MORFs) for studying magnetic interactions with transition-metal ions.^10,11^ More recently, a Zn(ii)-based radical coordination polymer has been shown to exhibit both luminescence and magnetoluminescence,^12,13^ highlighting the potential of radical-containing frameworks as multifunctional materials.

Multivariate (mixed-linker) MOFs have emerged as a powerful platform for tailoring the properties of crystalline materials by integrating chemically distinct linkers within a single framework.^14–16^ This strategy enables the systematic modulation of electronic and optical properties while preserving the underlying crystal structure.^17^ For example, a multivariate MORF incorporating both radical and non-radical PTM tricarboxylate (PTMTC) linkers, which differ in their electronic structure, was recently shown to preserve the optical, magnetic, and electronic properties of the radical within the framework.^18^

To further demonstrate the potential of mixed-linker MORFs for modulating the properties of radical-based frameworks, herein we report an isostructural series of multivariate MORFs incorporating both radical (PTMTC^R^) and non-radical (PTMTC^NR^) PTM tricarboxylate linkers within the same crystalline structure. By simply varying the ratio of the two linkers, the radical content can be tuned from *ca.* 40 to 80% while preserving the framework architecture. Single-crystal X-ray diffraction unambiguously reveals the statistical co-occupancy of radical and non-radical linkers at the same crystallographic site in MORF-40, whereas complementary spectroscopic and microscopic techniques demonstrate the modulation of the radical content across the MORF series. This mixed-linker strategy provides a versatile platform for tuning the electronic, magnetic, and optical properties of radical-based MOFs.

## Results and discussion

### Synthesis of multivariate MORFs

Building on our previous work on the PTMTC-based MORF,^18^ we sought to control the incorporation of radical and closed-shell linkers within a single crystalline framework. To this end, the closed-shell PTMTC^NR^ and open-shell PTMTC^R^ linkers (Scheme 1) were synthesised following our previously reported procedures with minor modifications (see the SI for details).^18^ Single crystals of MORF-40, MORF-60, and MORF-80 were obtained by slow diffusion of an ethanol/water solution of Zn(Cl)_2_ into a pyridine/ethanol solution containing PTMTC^R^ and PTMTC^NR^ in molar ratios of 4 : 6, 6 : 4, and 8 : 2, respectively (see SI). The crystallisation mixtures were left undisturbed at 30 °C for two weeks, affording single crystals suitable for structural characterisation. This synthetic strategy afforded a family of mixed-linker MORFs with varied radical contents while preserving the same framework structure (Scheme 2).

**Scheme 1:**
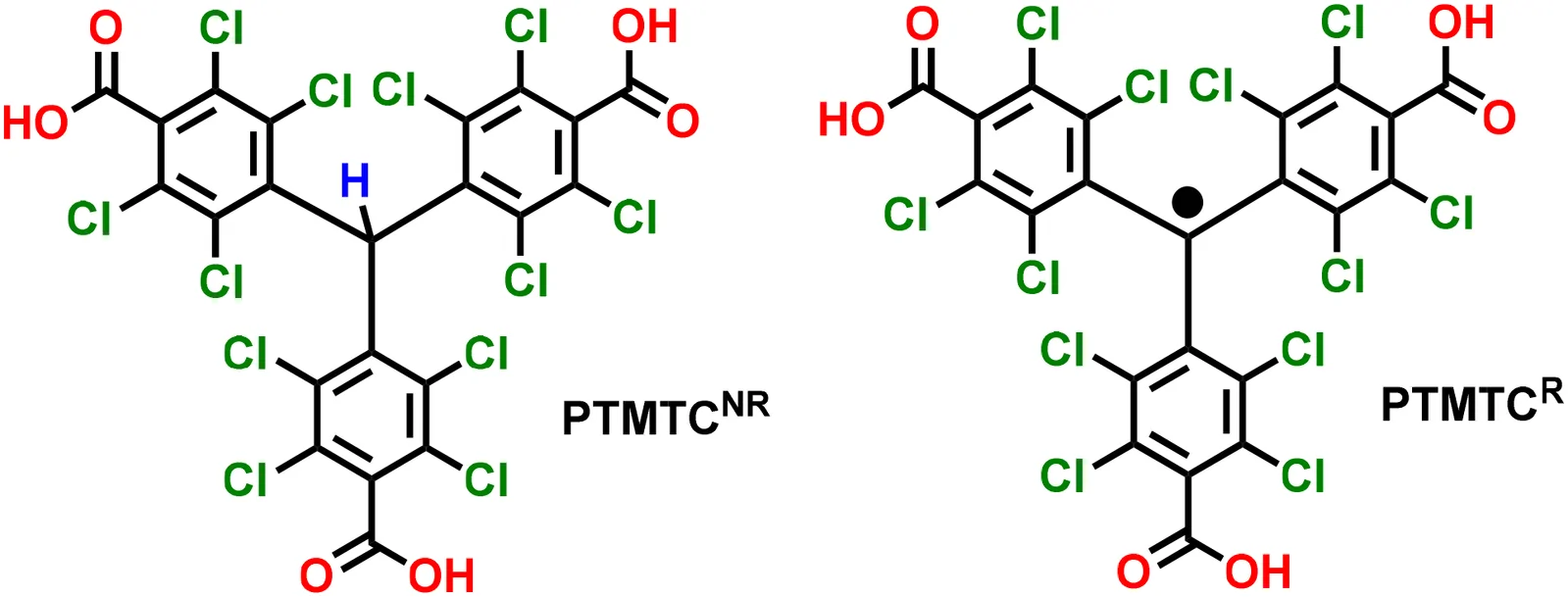
Molecular structures of PTMTC^NR^ and PTMTC^R^.

**Scheme 2:**
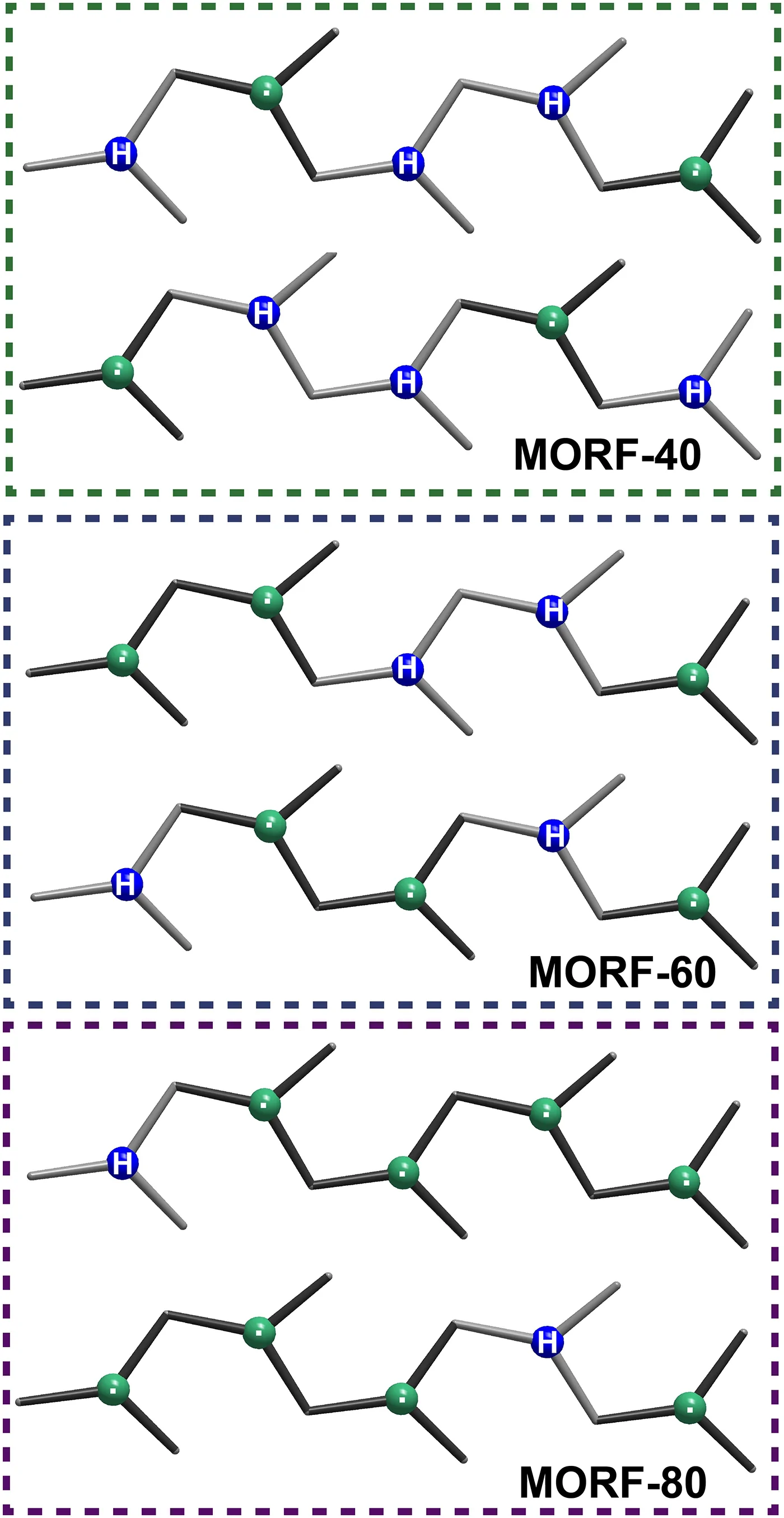
Schematic representation of the structures of MORF-40, MORF-60, and MORF-80 with different radical content.

### Single-crystal X-ray diffraction

Owing to the limited quality of the obtained crystals, a full single-crystal X-ray diffraction refinement was only possible for MORF-40. The crystal structure unambiguously confirms the coexistence of both PTMTC^R^ and PTMTC^NR^ linkers within the framework. MORF-40 crystallises in the centrosymmetric space group *P*2_1_/*c*, with the asymmetric unit comprising one PTMTC linker, two Zn(ii) centres, one coordinated chloride ion, four coordinated pyridine ligands, and 0.85 molecules of crystallisation pyridine (Fig. 1), corresponding to the formula [Zn_2_(PTMTC^NR^)_0.6_(PTMTC^R^)_0.4_(py)_4_Cl]·0.85py. The PTMTC linker is disordered, with the radical and non-radical species occupying essentially the same crystallographic position (Fig. 1 and Fig. S1). Refinement of the disorder converged to occupancies of 0.60 and 0.40 for the non-radical (PTMTC^NR^) and radical (PTMTC^R^) linkers, respectively (see SI). The radical component was identified by the absence of electron density at the central carbon atom (C106), whereas the hydrogen atom of the non-radical –CH group was located from the difference Fourier map. Each PTMTC linker coordinates three Zn(ii) centres through monodentate (κ^1^-*O*) carboxylate coordination. The two crystallographically independent Zn(ii) centres both adopt distorted tetrahedral geometries: one is coordinated by one PTMTC linker, two pyridine ligands, and one chloride ion, whereas the second is coordinated by two PTMTC linkers and two pyridine ligands.

**Fig. 1:**
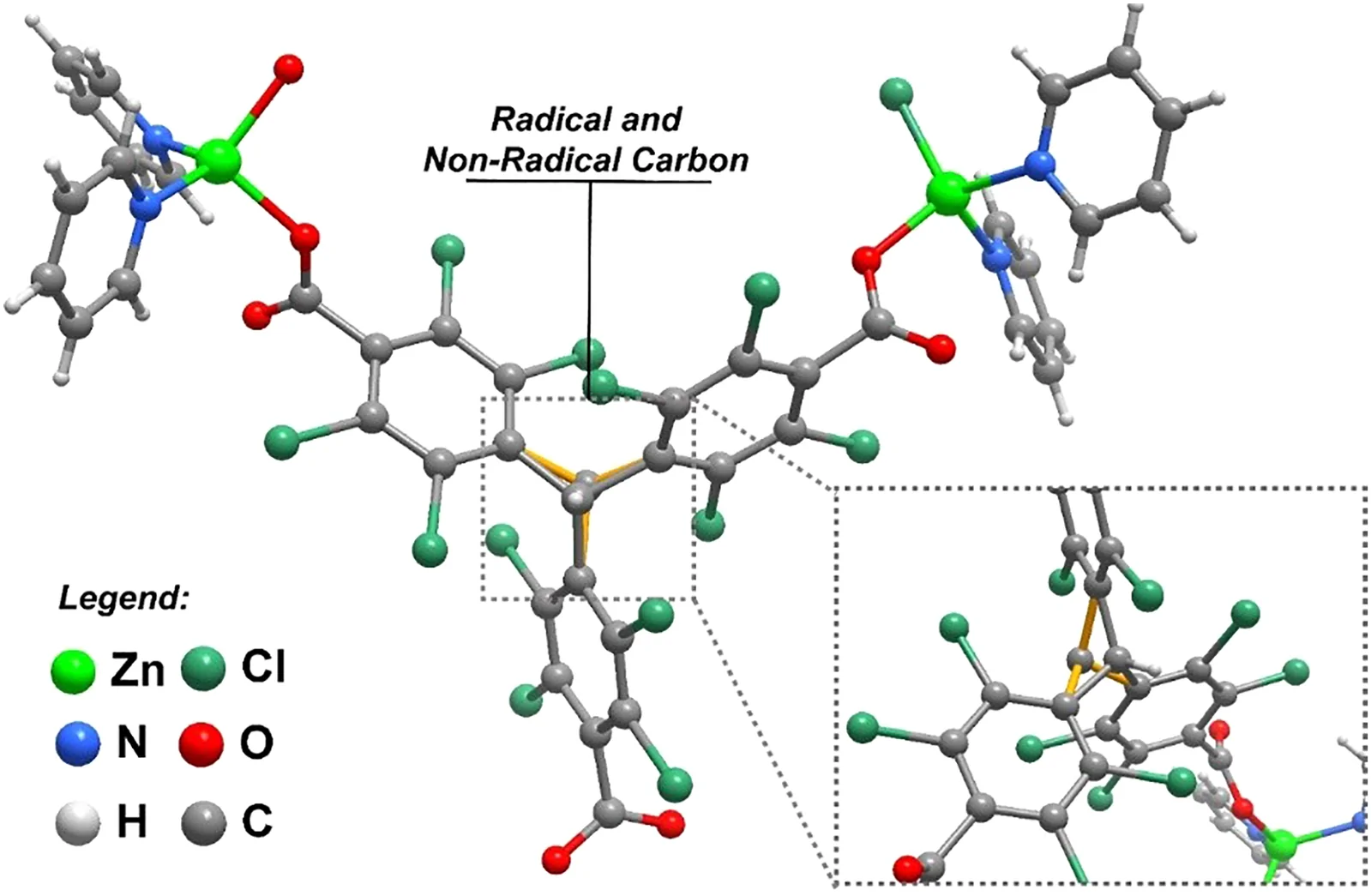
Schematic representation of the asymmetric unit of [Zn_2_ (PTMTC^NR^)_0.6_(PTMTC^R^)_0.4_(py)_4_Cl]·0.85py (MORF-40), highlighting the coexistence of radical and non-radical linkers occupying the same crystallographic position. For clarity, the disorder of the PTMTC ligand and the crystallisation pyridine molecules has been omitted, with only the disorder associated with the central carbon atom shown.

These different coordination environments of the two Zn(ii) centres give rise to a one-dimensional coordination chain propagating along the [010] direction in a zig-zag arrangement (Fig. 2), with a Zn⋯Zn separation of 15.5 Å. Despite the large number of aromatic rings associated with the PTMTC and pyridine ligands, no significant π–π interactions are observed, and the crystal packing reveals weak C–H⋯O hydrogen-bonding interactions. The average C–C bond distance between the central carbon atom of the PTM moiety and the phenyl rings is *ca.* 1.51 Å for the PTMTC^R^ linker and *ca.* 1.52 Å for the PTMTC^NR^ linker. These intermediate values are consistent with the coexistence of both linker types within the crystal. For comparison, PTM non-radical molecules typically exhibit C–C bond lengths of *ca.* 1.55 Å, whereas PTM radical moieties display shorter C–C distances in the range 1.46–1.48 Å.^18–20^

**Fig. 2:**
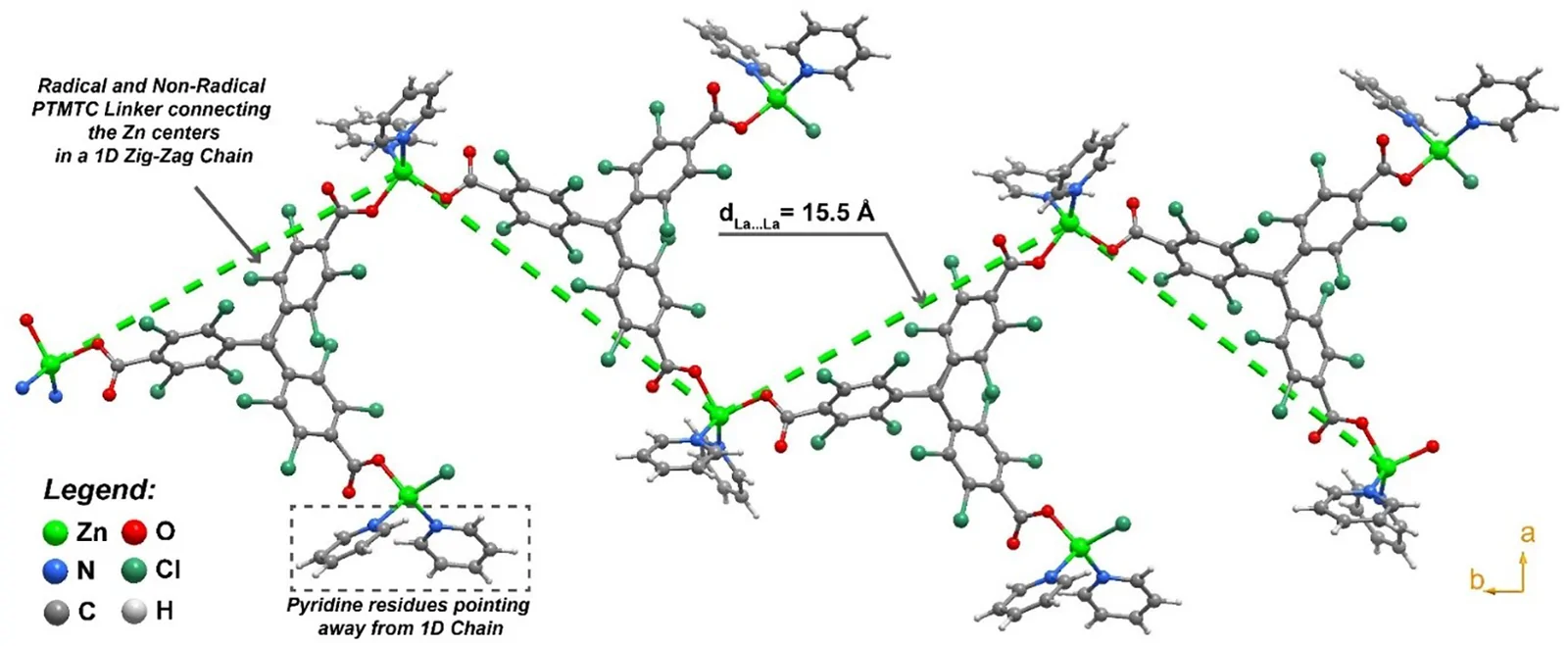
Schematic representation of the 1D coordination polymer [Zn_2_ (PTMTC^NR^)_0.6_(PTMTC^R^)_0.4_(py)_4_Cl]·0.85py (MORF-40), highlighting its zigzag chain with a Zn⋯Zn separation (*d*_Zn⋯Zn_) of 15.5 Å, viewed along the [001] direction. Crystallisation pyridine molecules have been omitted for clarity.

### Powder X-ray diffraction

The incorporation of increasing amounts of PTMTC^R^ linkers does not alter the overall crystal structure of the MORF family. Single-crystal X-ray diffraction reveals that the radical and non-radical linkers occupy the same crystallographic position within the framework, indicating that both species can be accommodated without perturbing the framework topology. Consistent with this observation, the powder X-ray diffraction (PXRD) patterns of MORF-40, MORF-60, and MORF-80 closely resemble one another with the main peak centred at 6.7° and are in excellent agreement with the simulated pattern calculated from the single-crystal structure of MORF-40 (Fig. 3). The absence of additional reflections or significant peak shifts is consistent with all members of the series being isostructural and retaining the same one-dimensional coordination framework irrespective of the radical/non-radical linker ratio. The close overlap of the experimental PXRD patterns further indicates that varying radical content does not induce detectable structural changes or the formation of secondary crystalline phases. These results demonstrate that the PTMTC^R^ radical linker can be incorporated over a broad compositional range while preserving both the crystallinity and structural integrity of the framework.

**Fig. 3:**
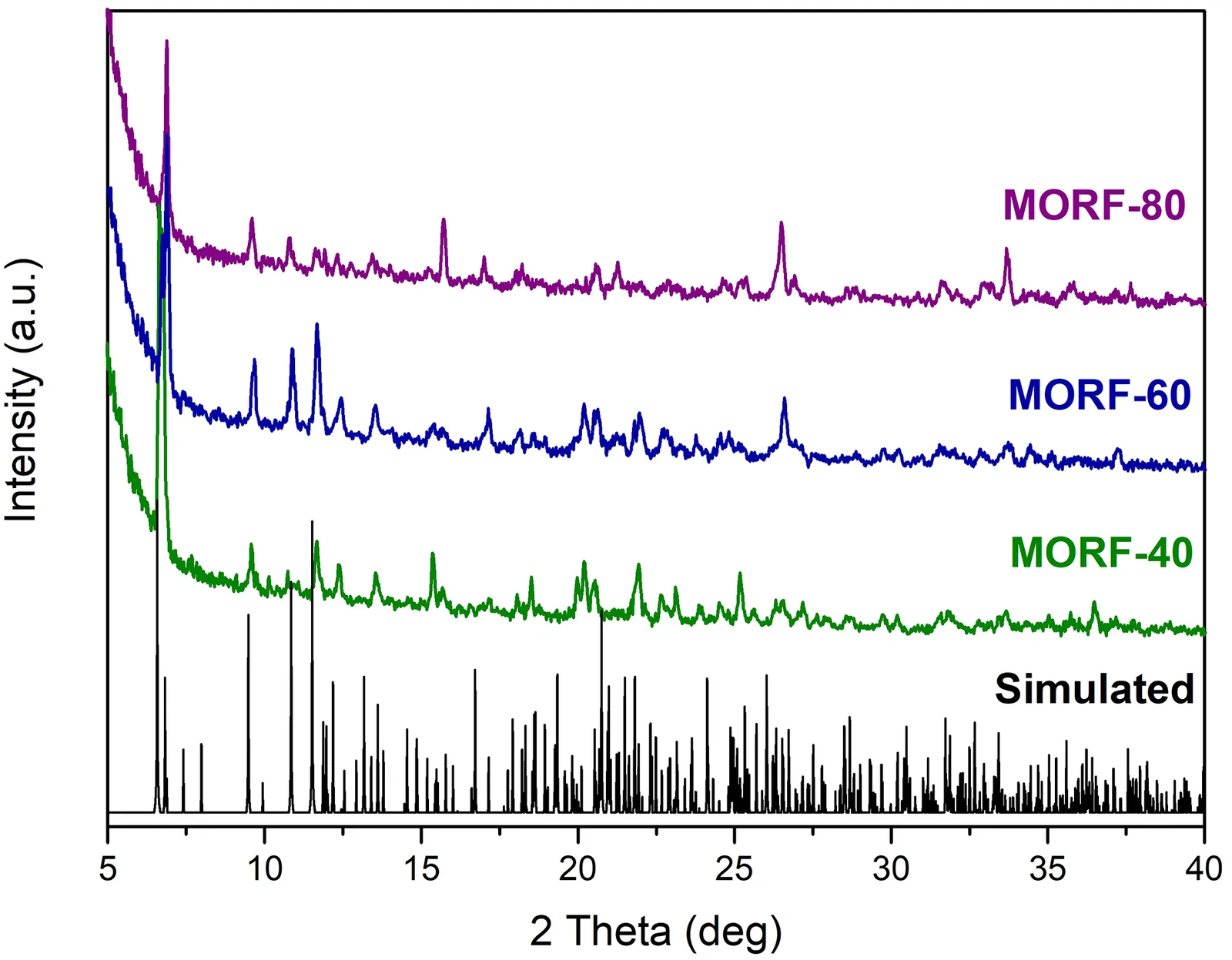
PXRD patterns of MORF-40 (experimental and simulated), MORF-60 (experimental), and MORF-80 (experimental).

### Electron paramagnetic resonance

The incorporation of PTMTC^R^ radical linkers within the MORF series was further investigated by electron paramagnetic resonance (EPR) spectroscopy (Fig. 4). The EPR spectrum of the free PTMTC^R^ ligand was recorded as a reference and exhibits an intense signal centred at *g* = 2.003, characteristic of PTM-based organic radicals.^19–21^ Crystals of MORF-40, MORF-60, and MORF-80 exhibit the characteristic EPR signal of the PTMTC^R^ radical linker. When measured using identical masses of material under the same experimental conditions, the signal intensity increases progressively from MORF-40 to MORF-80, in agreement with the increasing proportion of radical linkers incorporated into the framework. Notably, EPR quantification^22^ revealed 0.24, 0.56, and 0.73 spins per unit cell for MORF-40, MORF-60, and MORF-80, respectively, confirming the progressive increase in radical content across the MORF series (see SI).

**Fig. 4:**
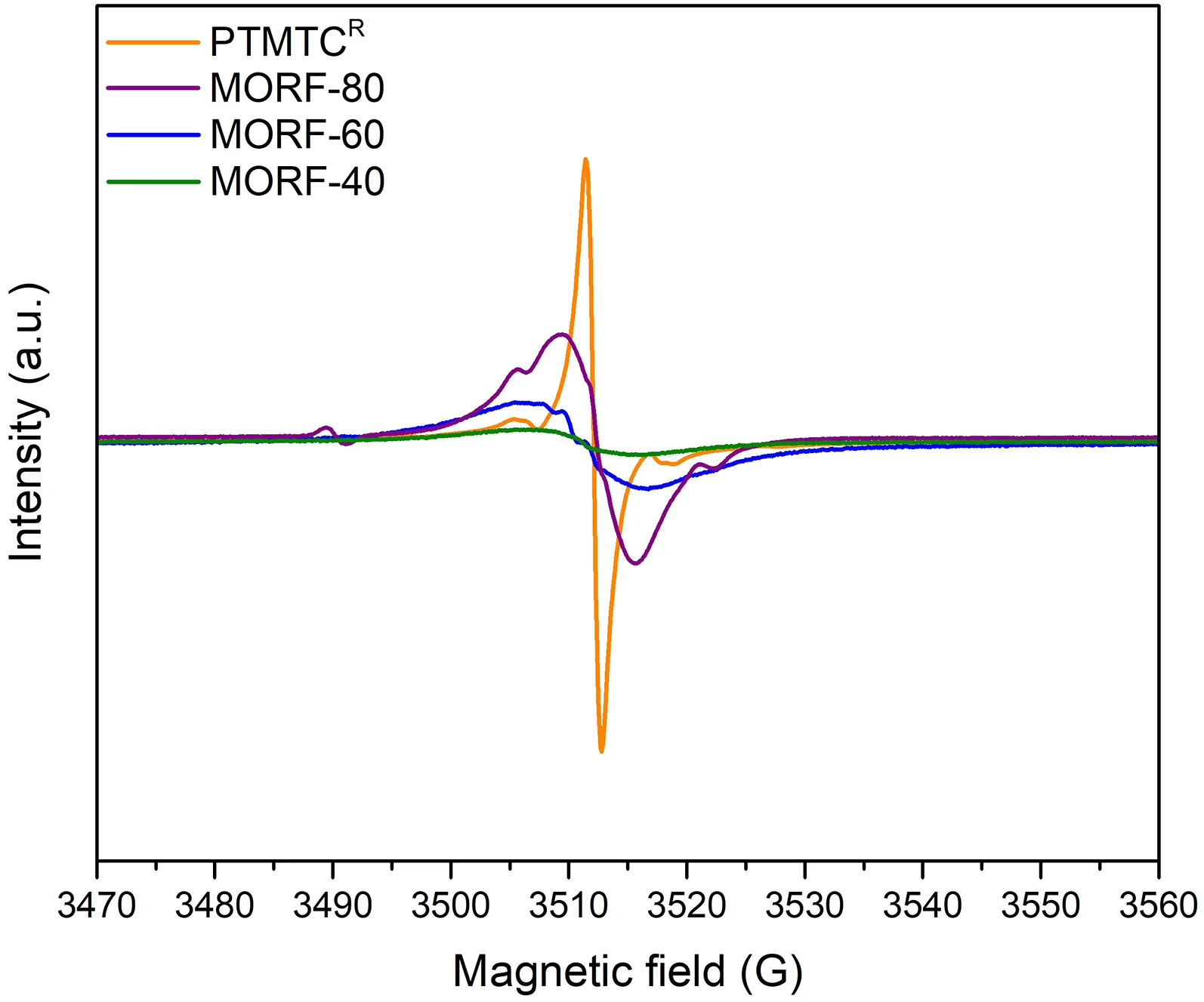
EPR spectra of PTMTC^R^, MORF-80, MORF-60, and MORF-40 crystals at 298 K.

### Raman spectroscopy

The incorporation of both PTMTC^R^ and PTMTC^NR^ linkers within MORF-40, MORF-60, and MORF-80 was further corroborated by Raman spectroscopy (Fig. 5). The spectrum of the PTMTC^NR^ non-radical ligand displays two bands at 1526 and 1556 cm^−1^ in the 1500–1600 cm^−1^ region, assigned to distinct aromatic C=C stretching modes of the chlorinated phenyl rings. In contrast, the PTMTC^R^ radical linker exhibits a single band at 1541 cm^−1^, reflecting the enhanced π-delocalisation and increased electronic equivalence of the three phenyl rings in the open-shell species, in agreement with previous reports on PTM-based radicals.^19,23^ Consistent with these observations, the Raman spectrum of MORF-80 crystals shows an intense band centred at 1531 cm^−1^, with a minor contribution at 1554 cm^−1^, in agreement with its higher PTMTC^R^ content. On the other hand, MORF-40 exhibits two Raman bands at 1525 and 1555 cm^−1^, consistent with the predominance of PTMTC^NR^ linkers (Fig. 5 and Fig. S2). Peak-fitting and deconvolution of the Raman bands in the 1500–1600 cm^−1^ region further support the progressive change in their relative composition across the MORF series (Fig. S2). Notably, an additional Raman band at 1612 cm^−1^, absent in both the PTMTC^NR^ and PTMTC^R^ ligands, emerges upon framework formation. This feature is tentatively assigned to a coupled asymmetric carboxylate stretching [*ν*_as_(COO^−^)] and aromatic *ν*(C=C) vibration associated with Zn–carboxylate coordination, in agreement with previous Raman studies of Zn–carboxylate MOFs.^24,25^

**Fig. 5:**
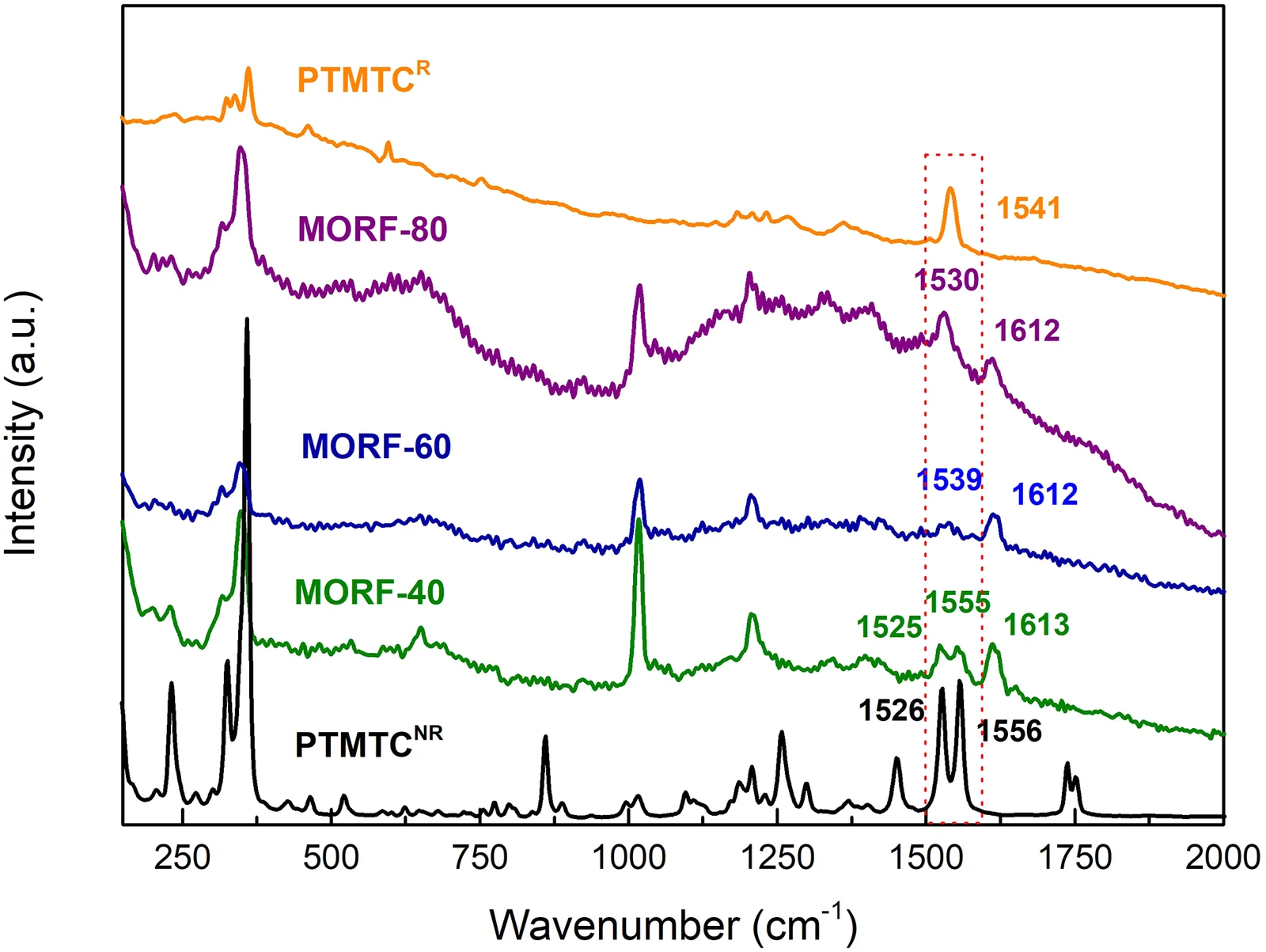
Raman spectra (*λ* = 785 nm) of PTMTC^R^, PTMTC^NR^, MORF-80, MORF-60, and MORF-40 in the solid state at 298 K.

### Kelvin probe force microscopy

Kelvin probe force microscopy (KPFM) measurements were performed on single crystals of MORF-40, MORF-60, and MORF-80 (see SI for experimental details). PTM radical and non-radical derivatives have been reported to exhibit distinct polarizabilities, which can lead to shifts in the work function.^18,26,27^ As a reference, the KPFM surface potential distribution of the parent framework containing exclusively PTMTC^NR^ non-radical linkers (PTMTC^NR^–Zn MOF)^18^ exhibits a unimodal profile centred at −0.89 V (Fig. 6a), in agreement with our previous report.^18^ Upon incorporation of PTMTC^R^ linkers, the potential distribution evolves into a bimodal profile (Fig. 6a–d), consistent with the coexistence of radical and non-radical linkers within the framework, as previously observed.^18^ It should be noted that KPFM provides only qualitative information on the local distribution and electronic behaviour of the two linker populations, as the measurements probe the surface of a number of crystals.

**Fig. 6:**
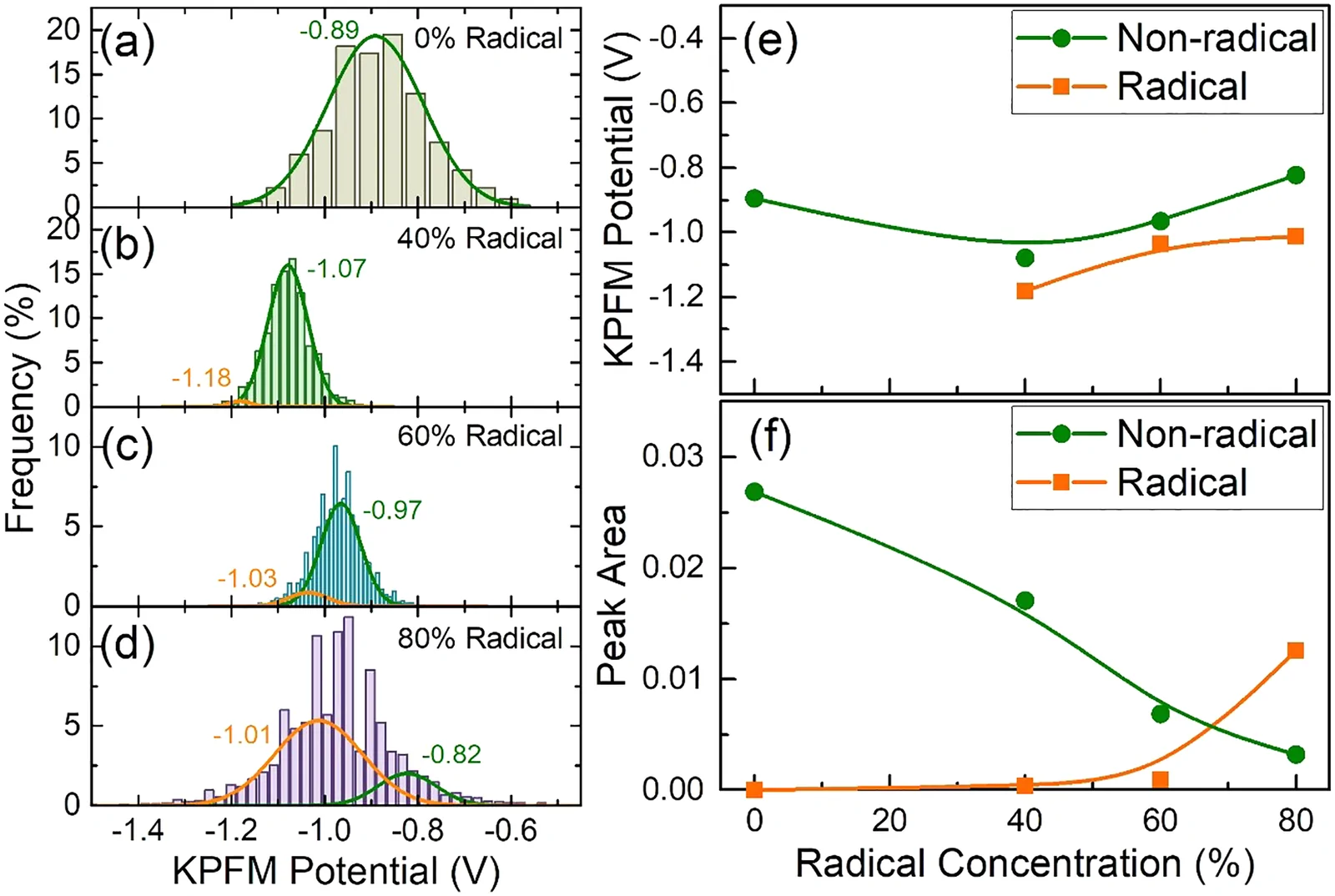
Distributions of KPFM potential over the surface of the multivariate MORF crystals with different content of radicals: (a) 0% (PTMTC^NR^–Zn MOF), (b) 40% (MORF-40), (c) 60% (MORF-60), and (d) 80% (MORF-80). Solid curves show the histogram fitting with the Gauss function. Radical content of (e) KPFM potential and (f) area of the peaks for both radical and non-radical phases.

The positions and relative areas of the two peaks assigned to the PTMTC^NR^ and PTMTC^R^ linker populations exhibit a non-linear dependence on the nominal NR/R ratio (Fig. 6e and f). For the MORF-40, the PTMTC^NR^-related peak shifts by −0.18 V, from −0.89 to −1.07 V, whereas further increasing the radical content causes the peak to shift towards higher potentials (Fig. 6e). A second peak, assigned to the radical linker population, emerges at lower potentials and displays a similarly non-linear evolution.

The relative peak areas provide a qualitative estimate of the linker composition within the crystals. The PTMTC^NR^ contribution, which dominates at low radical contents, progressively decreases with increasing PTMTC^R^ incorporation and becomes nearly negligible for the framework containing 80% radical linkers (Fig. 6f).

### Fluorescence microscopy

To investigate the influence of radical incorporation on the photophysical properties of the MORF series, all samples were analysed by confocal fluorescence microscopy using the average hyperspectral data for analysis (Fig. 7 and Fig. S3 and S4). Notably, the free PTMTC^R^ radical ligand exhibits an intense emission band at 625 nm, whereas the PTMTC^NR^ non-radical ligand is essentially non-emissive.^18^ The d^10^ closed-shell electronic configuration of Zn(ii) also minimises quenching of radical luminescence. As shown in Fig. 7, excitation at 465 nm produces a broad emission centred at 605 nm for MORF-80. The intensity of this emission band decreases progressively with decreasing radical content from MORF-80 to MORF-40. The emission band observed for the radical-containing MORFs is consistent with the characteristic photoluminescence of PTM-based radicals, which typically exhibit broad orange-red emission in the 600–630 nm region.^19,28^ Analysis of the confocal fluorescence data confirms a systematic increase in emission intensity with increasing PTMTC^R^ radical content from MORF-40 to MORF-80 (Fig. S5). Although the dependence is not strictly linear, the observed trend is consistent with the incorporation of increasing amounts of PTMTC^R^ linkers into the framework while preserving the crystalline structure.

**Fig. 7:**
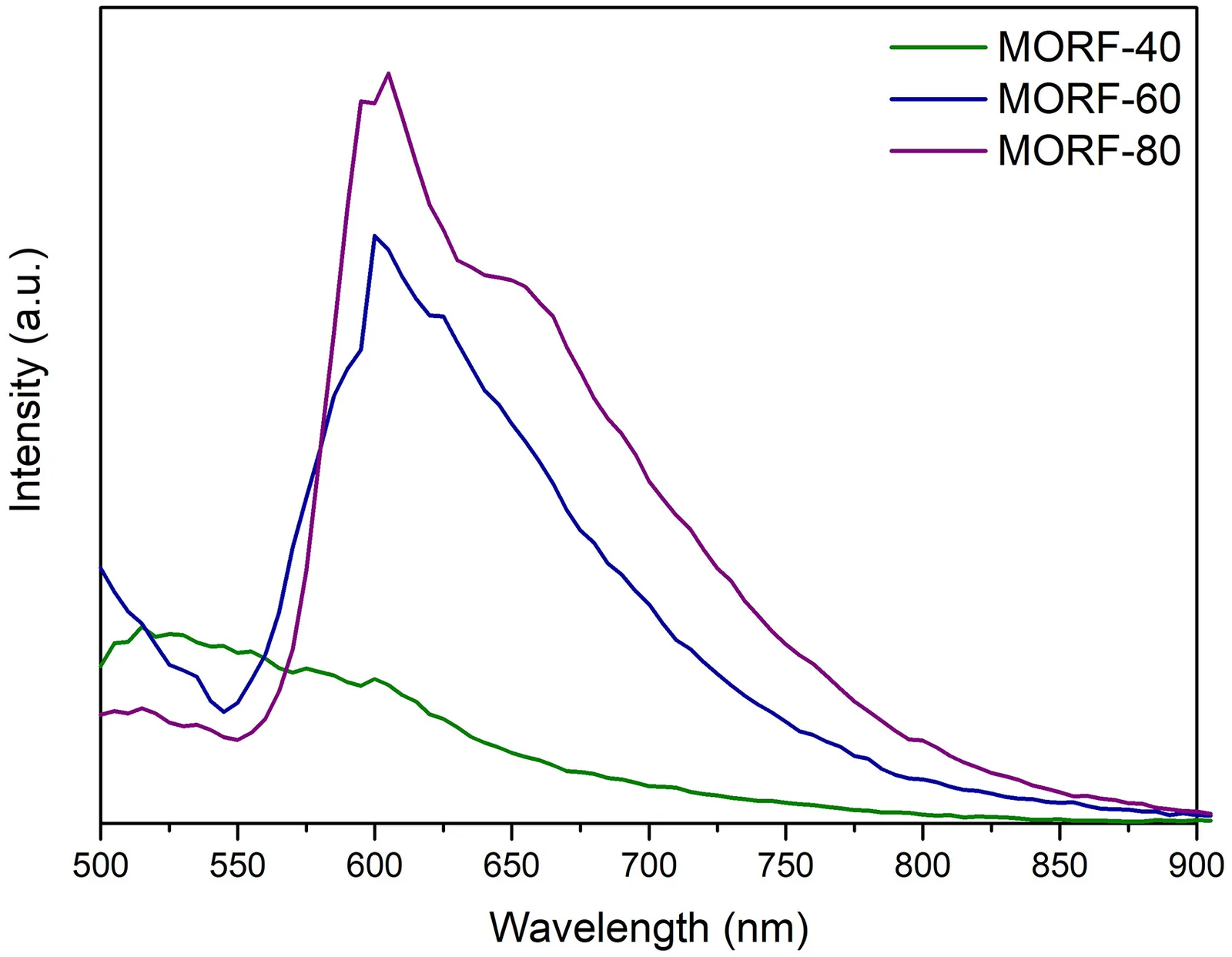
Emission spectra of single crystals of MORF-80, MORF-60, and MORF-40 recorded at 298 K upon 465 nm excitation using a confocal microscope.

## Conclusions

In summary, we have developed a family of multivariate MORFs in which the ratio of radical (PTMTC^R^) and non-radical (PTMTC^NR^) linkers can be systematically modified while preserving the same framework architecture. Single-crystal X-ray diffraction directly demonstrates the statistical co-occupancy of both linkers at the same crystallographic site in MORF-40, whereas PXRD, EPR, Raman spectroscopy, KPFM, and fluorescence microscopy demonstrate the successful modulation of the radical content across the series. The ability to tune the content of open-shell linkers within an isostructural framework enables the modulation of the electronic and photophysical properties without compromising crystallinity or framework integrity. In particular, the systematic evolution of EPR, KPFM, and fluorescence measurements corroborates that the radical content can be used as a design strategy for multivariate MORFs, enabling the development of crystalline materials with programmable optical, electronic, and magnetic functionalities. We anticipate that extending this approach to other persistent organic radicals, framework topologies, and metal centres will further expand its scope. In particular, combining PTMTC^R^ radical linkers with high-valent metal centres (*e.g.*, Zr^4+^, Ti^4+^) could potentially enhance the thermal and chemical stability of these materials, while retaining their redox and magnetic properties. Such developments could broaden the potential of these materials towards applications in energy storage, electrocatalysis, optoelectronics and spintronics, among others.

## Experimental

### General methods and materials

All reagents and solvents were of analytical grade and were purchased from Sigma-Aldrich or TCI. Unless otherwise stated, they were used as received without further purification. The synthesis and characterisation of the PTMTC^NR^ and PTMTC^R^ ligands, together with the preparation of the multivariate MORFs, are described in the SI.

### Single-crystal X-ray diffraction

Single crystals of MORF-40 were manually harvested from the crystallisation vial and immersed in highly viscous FOMBLIN Y perfluoropolyether vacuum oil (LVAC 140/13, Sigma-Aldrich) to avoid degradation caused by the evaporation of the solvent. Crystal was mounted on MiTeGen MicroLoops, typically with the help of a Stemi 2000 stereomicroscope equipped with Carl Zeiss lenses. Crystal data for MORF-40 was collected at 150 K on a RIGAKU XtaLAB Synergy-i equipped with a Mo Kα (*λ* = 0.71073 Å) PhotonJet-i microsource, a HyPix3000 detector controlled by the CrysAlisPro2 software and equipped with an Oxford Cryosystems Series 800 cryostream. Diffraction images were processed using the CrysAlisPro software and the data were corrected for absorption by the multiscan absorption correction using spherical harmonics implemented in SCALE3 ABSPACK scaling algorithm. The occupancies of the radical (PTMTC^R^) and non-radical (PTMTC^NR^) linkers were initially refined independently against the diffraction data, converging to values in the range of 30–40% and 60–70%, respectively. As these values were consistent with the nominal linker ratio used in the synthesis, the final refinement was performed by constraining the occupancies to 0.40 for PTMTC^R^ and 0.60 for PTMTC^NR^.

### Powder X-ray diffraction (PXRD)

PXRD patterns were recorded using a Rigaku MiniFlex 600-C X-ray diffractometer using Cu K*α*_1,2_ radiation (*λ*_1_ = 1.540598 Å; *λ*_2_ = 1.544426 Å). The X-ray tube was operated at a voltage of 40 kV and a current of 15 mA. Data were collected in the *ca.* 2° ≤ 2*θ* ≤ 50° range with a 0.01° step.

### Electron paramagnetic resonance (EPR)

Spectra were recorded using an X-Band (9.4 GHz) Bruker ELEXSYS E-500 spectrometer with a ST8911 microwave cavity, a Bruker variable temperature unit, and a field frequency lock system (Bruker ER 033 M).

### Raman spectroscopy

Raman spectra of MORF single crystals were recorded using a WITec alpha300R confocal Raman microscope (785 nm).

### Kelvin probe force microscopy

KPFM measurements were performed at room temperature in a rarefied atmosphere (air pressure 3 × 10^−2^ Torr) using Park NX HiVac microscope (Park Systems). A supersharp AFM probe SSS-NCHR10 (Nanosensors, Switzerland) with the tip curvature radius below 2 nm and the resonance frequency of 249.7 kHz has been used. The amplitude of the free vibrations was set at 12 nm and non-contact set point was 80% of the free vibrations (9.6 nm) that corresponds to the tip–sample distance of about 16 nm. KPFM measurements were done in a single-pass mode with AC voltage of amplitude 1.5 V and frequency 17 kHz. No DC voltage bias was applied between the tip and the sample. The measured KPFM signal was analysed by a built-in lock-in amplifier with time constant 1 ms and sensitivity 0.1 V.

### Fluorescence microscopy

Confocal fluorescence microscopy measurements were performed using an Andor Dragonfly high speed confocal microscope (Oxford Instruments) equipped with a 465 nm excitation source. Emission spectra were collected from individual crystals at room temperature.

### Synthesis of multivariate MORFs

PTMTC^R^/PTMTC^NR^ mixtures (4/6, 6/4, and 8/2 ratio; 8 mg, 0.0101 mmol) and zinc chloride (ZnCl_2_, 2 mg, 0.0147 mmol) were weighed and placed in 4 mL vials. The mixtures were dissolved in 2 mL of an ethanol/water mixture (3 : 1, v/v), followed by the careful addition of 2 mL of an ethanol/pyridine mixture (7 : 1, v/v). The resulting solutions were left undisturbed to slowly evaporate at room temperature. After 15–20 days, several red single crystals were obtained, corresponding to MORF-40, MORF-60, and MORF-80, respectively.

## Author contributions

T. Z., G. V., and R. F. M. synthesised and characterised the materials. R. F. M. carried out the solution and refinement of the structure from the single crystal data. P. Z. carried out the KPFM measurements. R. F. M. and M. S. conceived the research and prepared the manuscript; all authors made comments on the manuscript.

## Conflicts of interest

There are no conflicts to declare.

## Supplementary Material

QM-OLF-D6QM00770H-s001

## Data Availability

Data for this article, including single crystal X-ray diffraction, PXRD, EPR, Raman, KPFM, and fluorescence microscopy data are available at Zenodo at https://doi.org/10.5281/zenodo.21914368. Supplementary information (SI): general information, synthetic procedures, single crystal X-ray diffraction, EPR, Raman spectroscopy, KPFM, and fluorescence microscopy. See DOI: https://doi.org/10.1039/d6qm00770h. CCDC 2577767 contains the supplementary crystallographic data for this paper.^29^
