# Supplementary material for: Tuning trityl radical content in multivariate metal–organic radical frameworks
Source: Mater Chem Front. 2026 Sep 30. Online ahead of print. doi: 10.1039/d6qm00770h (PMC13644682; doi:10.1039/d6qm00770h)
Supplement: Supplementary file 1 [file QM-OLF-D6QM00770H-s001.pdf]

Supplementary Information (SI) for Materials Chemistry Frontiers.
This journal is © the Partner Organisations

Supporting Information

Tuning Trityl Radical Content in Multivariate Metal-Organic Radical Frameworks

Tianshu Zhang,[a] Gonçalo Valente,[b] Pavel Zelenovskii,[b] Ricardo F. Mendes,[a],* Manuel Souto[a,c],*

[a] *Centro Singular de Investigación en Química Biolóxica e Materiais Moleculares (CiQUS), Departamento de Química Física, Universidade de Santiago de Compostela, 15782 Santiago de Compostela, Spain*

[b] *CICECO-Aveiro Institute of Materials, University of Aveiro, 3810-393 Aveiro, Portugal*

[c] *Oportunius, Galician Innovation Agency (GAIN), 15702 Santiago de Compostela, Spain*

Table of contents

1. **General Information** S3
2. **Synthetic procedures** S4
3. **Single Crystal X-ray Diffraction** S9
4. **Electron Paramagnetic Resonance (EPR)** S11
5. **Raman Spectroscopy** S13
6. **Kelvin Probe Force Microscopy** S13
7. **Fluorescence Microscopy** S14
8. **References** S16

1. General Information

All reagents and solvents employed in the synthesis were of high-purity grade and were purchased from Sigma-Aldrich Co. or TCI.

^{1}H, and ^{13}C NMR experiments were carried out using a Varian Mercury 300MHz NMR spectrometer. Chemical shift values are reported in ppm with the solvent resonance as the internal standard ($CHCl_3$: δ = 7.26 ppm for ^{1}H; DMSO: δ = 2.5 ppm for ^{1}H).

MALDI-TOF-MS measurements were performed on an Autoflex Bruker spectrometer using negative mode.

Powder X-ray diffraction (PXRD) patterns were recorded using a Rigaku MiniFlex 600-C X-ray diffractometer using Cu Kα radiation (λ = 1.54056 Å). The X-ray tube was operated at a voltage of 40 kV and a current of 15 mA. Data were collected in the ca. 2º ≤ 2θ ≤ 40º range.

EPR spectra were recorded using an X-Band (9.4 GHz) Bruker ELEXSYS E-500 spectrometer with a ST8911 microwave cavity, a Bruker variable temperature unit, and a field frequency lock system (Bruker ER 033 M).

Raman spectra of MORF single crystals were recorded using a WITec alpha300R confocal Raman microscope (785 nm).

Confocal fluorescence microscopy measurements were performed using an Andor Dragonfly high speed confocal microscope (Oxford Instruments) equipped with a 465 nm excitation source. Emission spectra were collected from individual crystals at room temperature.

2. Synthetic procedures

Tris(2,3,5,6-tetrachlorophenyl)methane (**1**) was synthesized via Friedel-Crafts alkylation of 1,2,4,5-tetrachlorobenzene with $CHCl_3$ in the presence of $AlCl_3$. Then the reaction of **1** with *n*-BuLi and tetramethylethylenediamine (TMEDA) in anhydrous THF at low temperature formed the corresponding trianion that subsequently reacted with ethyl chloroformate to give the perchlorotriphenylmethyl triester **2**. Hydrolysis of ester **2** with concentrated sulfuric acid at 90 °C yielded the non-radical perchlorotriphenyl tricarboxylic acid **PTMTC^{NR}** as a white powder. Finally, subsequent deprotonation of **PTMTC^{NR}** with NaOH followed by oxidation of the corresponding carbanion with I_2 afforded **PTMTC^{R}** as a red powder, whose formation was monitored by UV-vis spectroscopy.

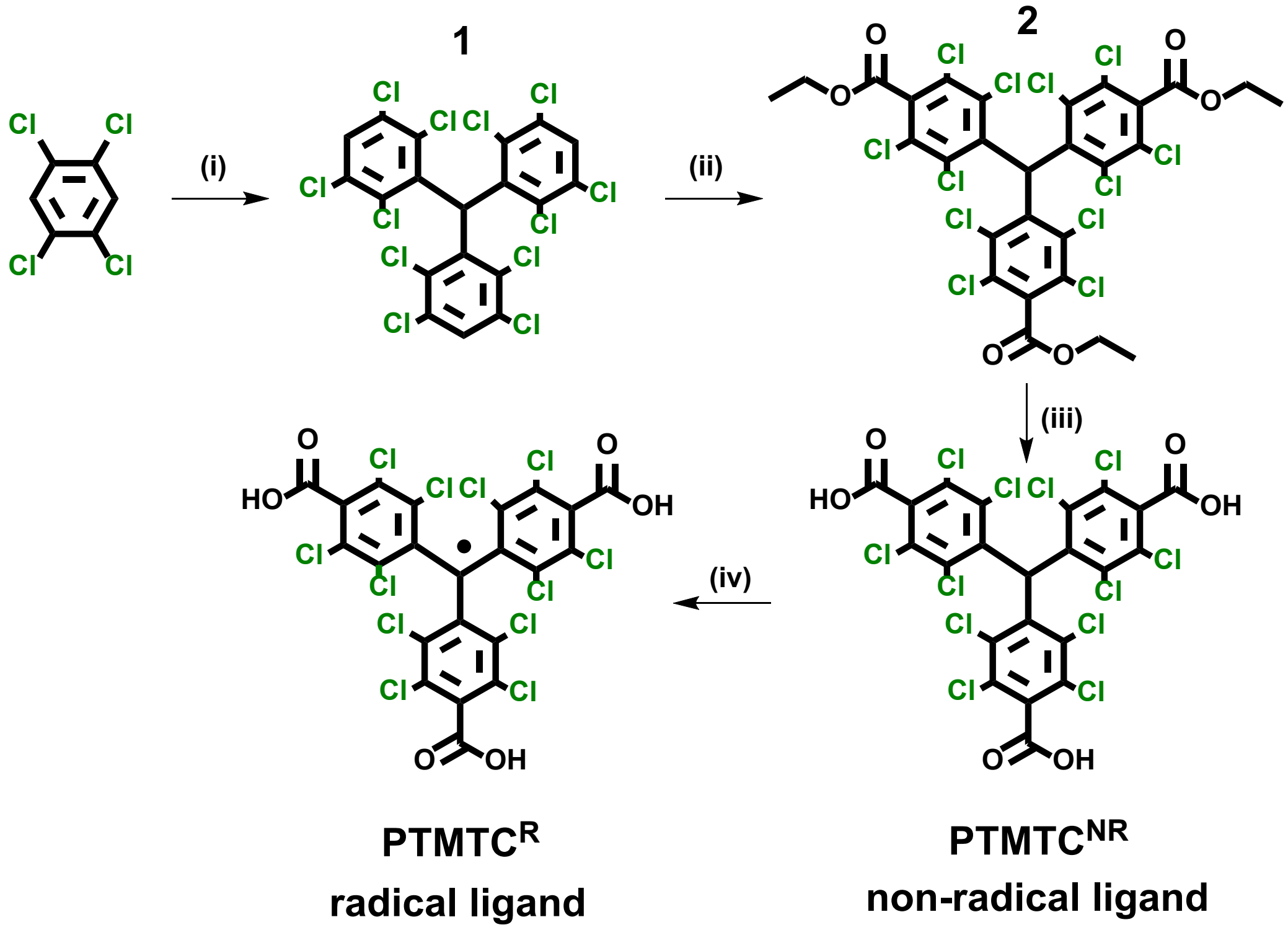

Scheme S1. Synthesis of **PTMTC^{R}**. (i) $AlCl_3$, $CHCl_3$, 165ºC, 24h, 61%; (ii) TMEDA, *n*-BuLi, THF, -78ºC, 1h; then ethyl chloroformate, 77%; (iii) conc. H_2SO_4, 90ºC, 12h, 69%; (iv) NaOH, DMSO, RT, 72h, then I_2, 44%.

2.1. Synthesis of tris(2,3,5,6-tetrachlorophenyl)methane (**1**)

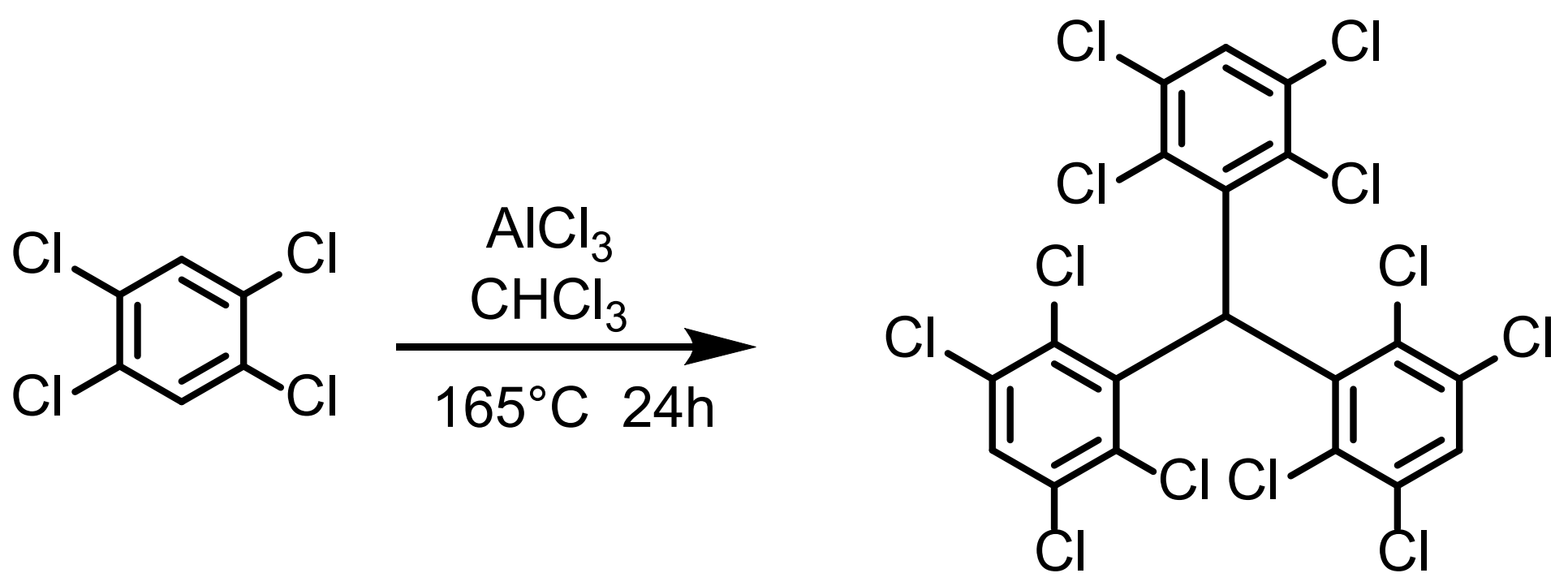

Scheme S2. Synthesis of tris(2,3,5,6-tetrachlorophenyl)methane (**1**).

Synthesis of tris(2,3,5,6-tetrachlorophenyl)methane (1). 1,2,4,5-Tetrachlorobenzene (6.4 g, 29.6 mmol) was mixed with aluminum chloride ($AlCl_3$, 1.11 g, 8.3 mmol) and $CHCl_3$ (0.67 mL) in a glass pressure reaction vessel equipped with a spiral tap. The reaction mixture was heated at 165 °C for 24 h. After cooling to 0 °C in an ice bath, hydrochloric acid (HCl, 1 M, 50 mL) was added. The mixture was sonicated and extracted with $CHCl_3$ (3 × 50 mL). The combined organic layers were washed sequentially with saturated aqueous $NaHCO_3$ (50 mL) and Milli-Q water (80 mL), and then dried over anhydrous $MgSO_4$. The drying agent was removed by filtration, and the solvent was evaporated under reduced pressure. The resulting red-brown solid was purified by column chromatography on silica gel using hexane as the sole eluent. Tris(2,3,5,6-tetrachlorophenyl)methane (**1**) was obtained as a white powder (61% yield). Characterization: ^{1}H-NMR (400 MHz, $CDCl_3$), δ (ppm): 7.65 (s, 3H), 6.98 (s, 1H). IR (cm^{-1}): 3116, 3068, 2929, 1728, 1547, 1410, 1386, 1346, 1322, 1238, 1198, 1161, 1097, 973, 864, 850, 780, 704, 688, 647, 623, 559, 539, 483.

2.2. Synthesis of tris(4-ethoxycarbonyl-2,3,5,6-tetrachlorophenyl)methane (2)

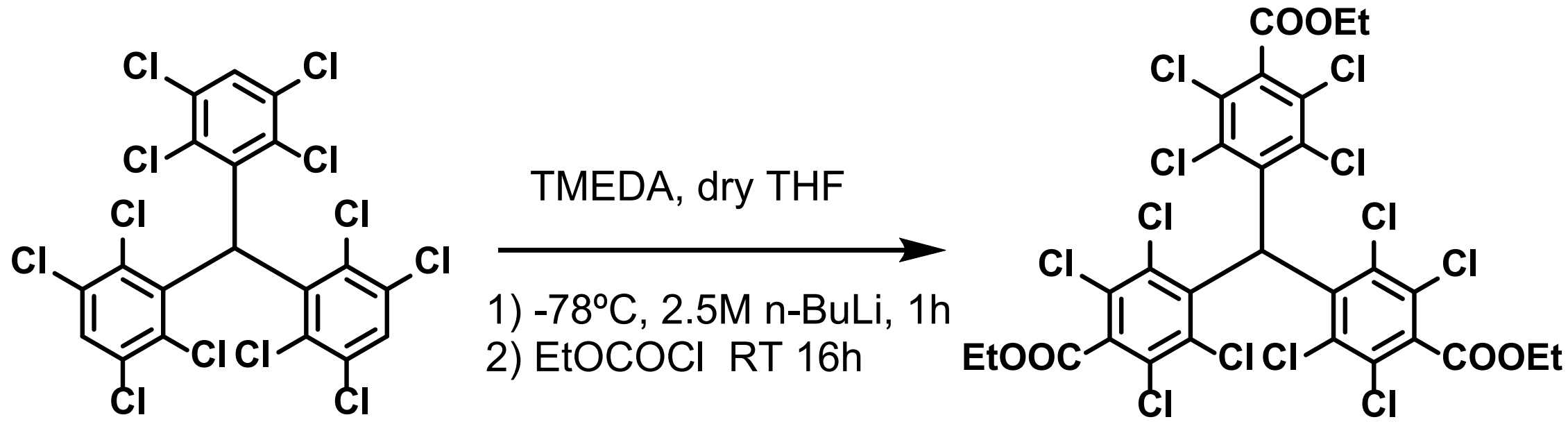

Scheme S3. Synthesis of tris(4-ethoxycarbonyl-2,3,5,6-tetrachlorophenyl)methane (**2**).

Synthesis of tris(4-ethoxycarbonyl-2,3,5,6-tetrachlorophenyl)methane (2). Tris(2,3,5,6-tetrachlorophenyl)methane (0.10 g, 0.15 mmol) and N,N,N′,N′-tetramethylethylenediamine (TMEDA, 0.23 mL, 1.5 mmol) were dissolved in anhydrous tetrahydrofuran (THF, 10 mL) under an inert atmosphere and cooled to −78 °C. A solution of *n*-BuLi (2.5 M in *n*-hexane, 0.80 mL, 2.0 mmol) was added in a single portion, and the reaction mixture was stirred at −78 °C for 1 h. Ethyl chloroformate (0.19 mL, 2.0 mmol) was then added, and the reaction mixture was allowed to warm to room temperature and stirred for 16 h. The reaction was subsequently quenched, and the solvent was removed under reduced pressure. The resulting residue was extracted with CH_2Cl_2 (3 × 10 mL), and the combined organic layers were washed with water (10 mL) and dried over anhydrous Na_2SO_4. After filtration, the solvent was removed under reduced pressure, and the crude product was purified by silica gel column chromatography using hexane/CH_2Cl_2 (1:1, v/v) as the eluent. Tris(4-ethoxycarbonyl-2,3,5,6-tetrachlorophenyl)methane (**2**) was obtained as a white solid (0.11 g, 77% yield). Characterization: ^{1}H-NMR (400 MHz, $CDCl_3$), δ (ppm): 7.02 (s, 1H), 4.49 (q, J = 7.1 Hz, 6H), 1.43 (t, J = 7.1 Hz, 12H). IR (cm^{-1}): 3026-2840 (w, C-H), 1750 (s, C=O), 1555, 1455, 1341, 1298, 1259, 1224, 1198 (s, C-O), 1113, 850 (s, C-Cl), 756, 651.

2.3. Synthesis of tris(4-carboxyl-2,3,5,6-tetrachlorophenyl)methane (PTMTC^{NR})

2 → **PTMTC^{NR}**

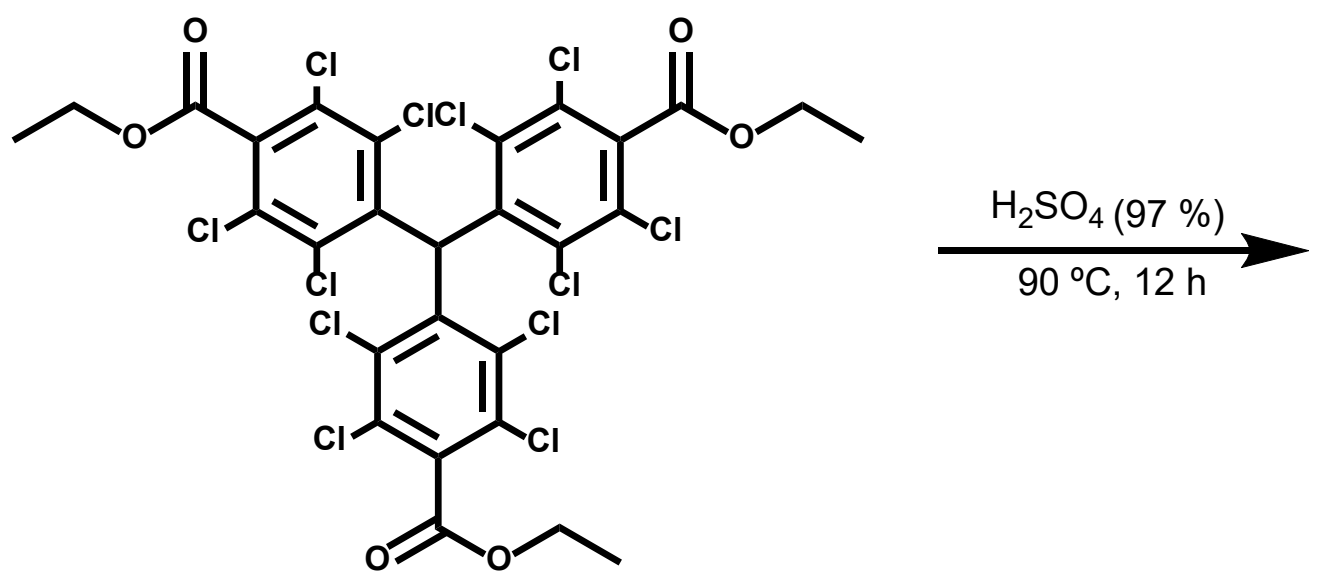

$C_{28}H_{16}Cl_{12}O_6$
873.86 gmol^{-1}

$C_{22}H_4Cl_{12}O_6$
789.70 gmol^{-1}

Scheme S4. Synthesis of tris(4-carboxyl-2,3,5,6-tetrachlorophenyl)methane (**PTMTC^{NR}**).

Synthesis of PTMTC^{NR}. Tris(4-ethoxycarbonyl-2,3,5,6-tetrachlorophenyl)methane (0.10 g, 0.12 mmol) was added to concentrated sulfuric acid (H_2SO_4, 97%, 15 mL), and the resulting mixture was heated at 90 °C for 18 h. After cooling to room temperature, the reaction mixture was poured onto ice, and the resulting aqueous phase was extracted with diethyl ether (Et_2O). The combined organic extracts were concentrated and subsequently extracted with an aqueous Na_2CO_3 solution. The resulting aqueous phase was acidified with 5 M HCl and extracted several times with Et_2O. The combined organic extracts were dried over anhydrous Na_2SO_4, filtered, and concentrated under reduced pressure. The crude product was dissolved in Et_2O and repeatedly precipitated by addition of hexane. **PTMTC^{NR}** was obtained as a white powder (0.173 g, 69% yield). Characterization: ^{1}H-NMR (400 MHz, DMSO-d$_6$), δ (ppm): 6.93 (s, 1H), 14.52 (s, 3H, Ar-COOH). ^{13}C-NMR (DMSO-d$_6$), δ (ppm): 164.28, 137.70, 137.51, 134.56, 133.84, 129.57, 128.61, 56.19. IR (cm^{-1}): 3020-2750 (w), 1672 (s, C=O), 1555, 1447, 1378, 1355, 1328, 1279, 1234, 1127, 885, 792, 715, 651, 526, 593.

2.4. Synthesis of tris(4-carboxyl-2,3,5,6-tetrachlorophenyl)methyl radical ($PTMTC^R$)

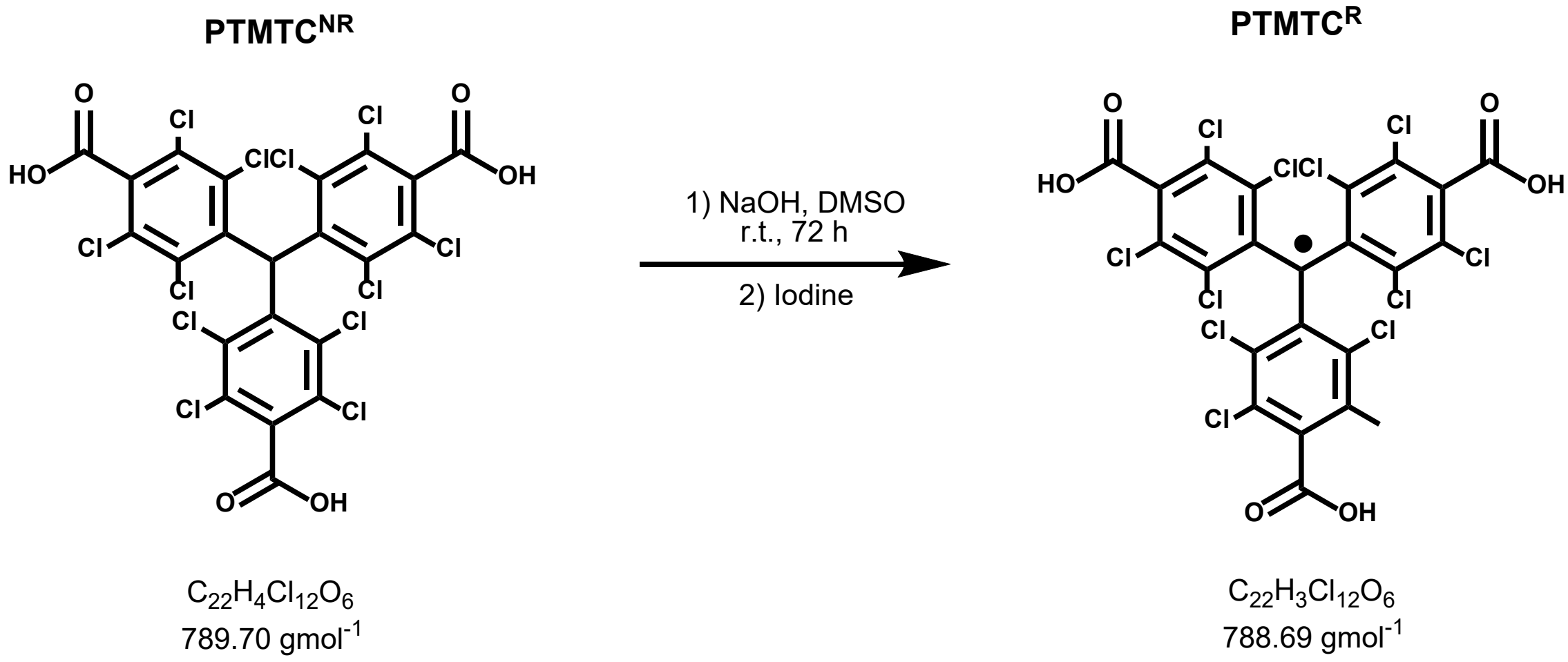

Scheme S5. Synthesis of radical tris(4-carboxyl-2,3,5,6-tetrachlorophenyl)methane radical (**$PTMTC^R$**).

Synthesis of $PTMTC^R$. Sodium hydroxide (0.175 g, 4.25 mmol) was dissolved in DMSO (5 mL), and the resulting solution was sonicated. **$PTMTC^{NR}$** (0.050 g, 0.0625 mmol) was then added, and the reaction mixture was stirred continuously in the dark for 72 h. The resulting mixture was filtered through cotton, and iodine (127 mg, 0.50 mmol) was added. The reaction mixture was left undisturbed in the dark and monitored by UV–vis spectroscopy until formation of the radical was confirmed by the disappearance of the characteristic absorption band of the anion at ca. 518 nm and the appearance of a new band at ca. 378 nm. The product was extracted with an aqueous solution of sodium hydrogen carbonate ($NaHCO_3$) and subsequently acidified with 12 M HCl. The resulting product was extracted with Et_2O and recrystallized from a mixture of Et_2O and *n*-pentane. **$PTMTC^R$** was obtained as a red powder (73 mg, 44% yield). Characterization: IR (cm^{-1}): 2925, 2852, 1695 (C=O), 1540, 1437, 1351, 1218, 1041, 722. UV-Vis (in $CHCl_3$) λ (nm) (ε [M^{-1} cm^{-1}]): 378 (17000). ESI-TOF-MS (*m*/*z*): 787.3 [M^-]. EPR (CH_2Cl_2, 300 K): g = 2.003.

2.5. Synthesis of multivariate MORFs

$PTMTC^R$/$PTMTC^{NR}$ mixtures (4/6, 6/4, and 8/2 ratio; 8 mg, 0.0101 mmol) and zinc chloride ($ZnCl_2$, 2 mg, 0.0147 mmol) were weighed and placed in 4 mL vials. The mixtures were dissolved in 2 mL of an ethanol/water mixture (3:1, v/v), followed by the careful addition of 2 mL of an ethanol/pyridine mixture (7:1, v/v). The resulting solutions were left undisturbed to slowly evaporate at room temperature. After 15–20 days, several red single crystals were obtained, corresponding to **MORF-40**, **MORF-60**, and **MORF-80**, respectively.

3. Single Crystal X-ray Diffraction

Single crystals of **MORF-40** were manually harvested from the crystallization vial and immersed in highly viscous FOMBLIN Y perfluoropolyether vacuum oil (LVAC 140/13, Sigma-Aldrich) to avoid degradation caused by the evaporation of the solvent.[1] Crystal was mounted on MiTeGen MicroLoops, typically with the help of a Stemi 2000 stereomicroscope equipped with Carl Zeiss lenses.

Crystal data for **MORF-40** was collected at 150(2) K on a RIGAKU XtaLAB Synergy-i equipped with a Mo Kα (λ = 0.71073 Å) PhotonJet-i microsource, a HyPix3000 detector controlled by the CrysAlisPro[2] software and equipped with an Oxford Cryosystems Series 800 cryostream. Diffraction images were processed using the CrysAlisPro software and the data were corrected for absorption by the multiscan absorption correction using spherical harmonics implemented in SCALE3 ABSPACK scaling algorithm.

The structure was solved using the algorithm implemented in SHELXT-2014/5,[3] which allowed the immediate location of almost all of the heaviest atoms composing the molecular unit of the four compounds. The remaining missing and misplaced non-hydrogen atoms were located from difference Fourier maps calculated from successive full-matrix least-squares refinement cycles on F^2 using the latest SHELXL from the 2018/3 release.[3] All structural refinements were performed using the graphical interface ShelXle.[4]

Hydrogen atoms bound to carbon were placed at their idealized positions using the *HFIX 43* instruction in SHELXL. Hydrogen atoms bound to carbon were placed at their idealized positions using the *HFIX 43* instruction in SHELXL. The disordered hydrogen atom bound to the central carbon atom of PTMTC molecule was located from difference Fourier maps and was included in the final structure model with fixed distance of 0.95 Å for C–H. These hydrogen atoms were included in subsequent refinement cycles with isotropic thermal displacements parameters (U_{iso}) fixed at 1.2 $\times U_{eq}$ of the parent atoms.

The occupancies of the radical ($PTMTC^R$) and non-radical ($PTMTC^{NR}$) linkers were initially refined independently against the diffraction data, converging to values in the range of 30–40% and 60–70%, respectively. As these values were consistent with the nominal linker ratio used in the synthesis, the final refinement was performed by constraining the occupancies to 0.40 for $PTMTC^R$ and 0.60 for $PTMTC^{NR}$.

The last difference Fourier map synthesis showed the highest peak (1.06 eÅ^{-3}) and the deepest hole (-1.48 eÅ^{-3}) located at 0.04 and 0.01 Å from C105 and C106, respectively. All structural drawings have been created using the software package Crystal Impact Diamond.

Crystallographic data (including structure factors) for the crystal structure reported in this manuscript have been deposited with the Cambridge Crystallographic Data Centre as supplementary publication data No. 2577767. Copies of the data can be obtained free of charge on application to CCDC, 12 Union Road, Cambridge CB2 2EZ, U.K. FAX: (+44) 1223 336033. E-mail: deposit@ccdc.cam.ac.uk.

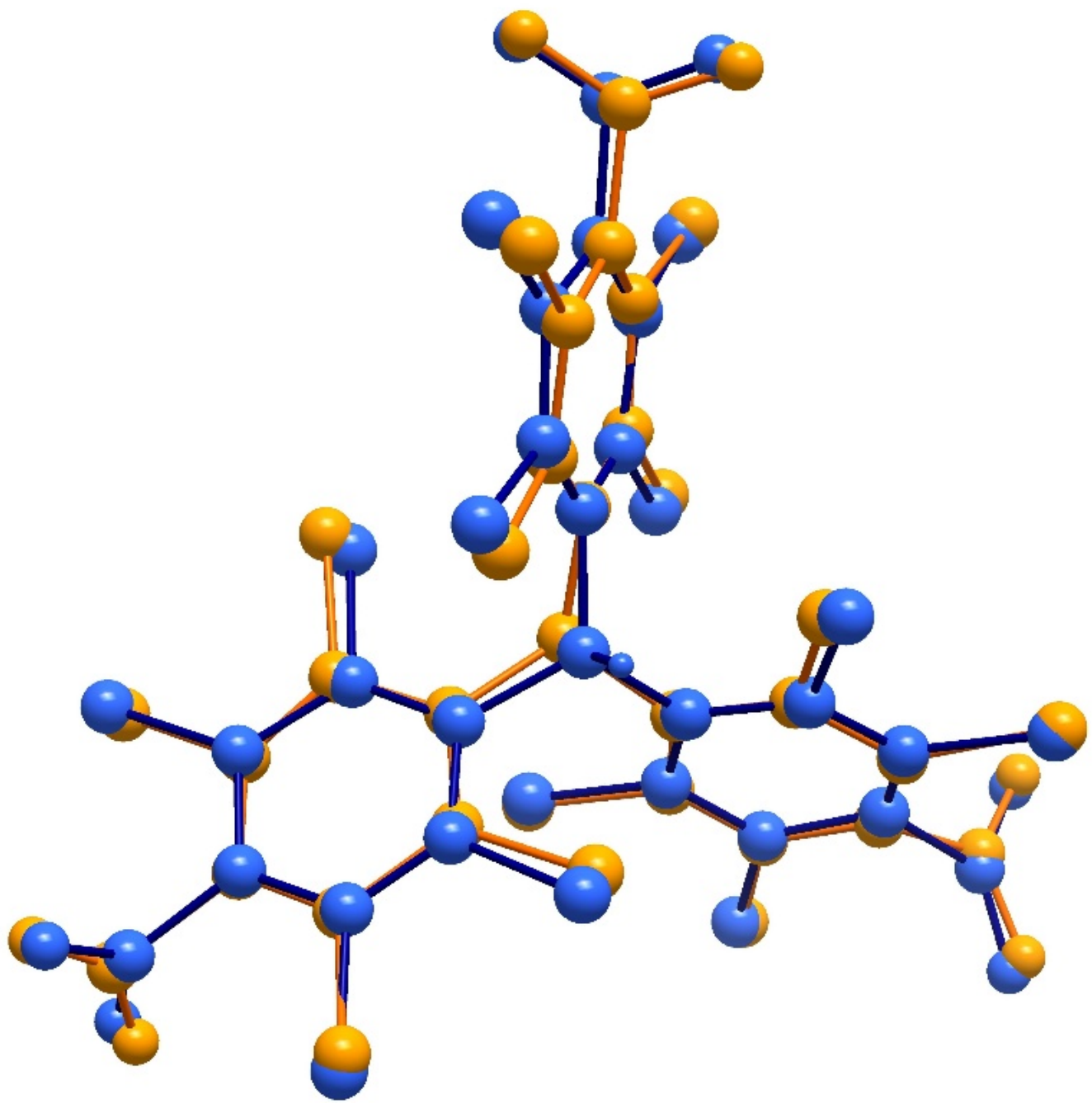

Figure S1. Schematic representation of the disorder present between the PTMTC non-radical (blue) and PTMTC radical (orange) present in $[Zn_2(HPTMTC)_{0.6}(PTMTC)_{0.4}(py)_4Cl] \cdot 0.85py$ (**MORF-40**).

4. Electron Paramagnetic Resonance (EPR)

The amount of sample introduced into the ESR tube, its position in the cavity, the microwave power, number of scans, conversion time and modulation amplitude, were optimized to achieve the best signal-to-noise ratio while maintaining signal linearity and avoiding saturation.

The doble integral (DI) of the ESR signal is related to the number of spins in the sample (n_s) through the following equation:

$$DI = C \times \sqrt{MWP} \times Q \times S(S+1) \times n_s$$

Where C is a constant which includes details of the experiment (time constant, spatial distribution of the microwave and modulation fields, modulation amplitude, etc), and MWP and Q are the microwave power and quality factor of the resonant cavity, respectively.

Therefore, comparing the DI of a standard with that of our sample, performed under the same conditions, results in:

$$n_{s(sample)} = n_{s(std)} \frac{DI(sample)}{DI(std)}$$

To ensure the reliability of the quantification, the EPR spin counting was calibrated using DPPH dispersed in quartz powder over a concentration range of 20–150 ppm. To minimize experimental error, all measurements were performed under identical conditions, including constant cavity filling factors, microwave power (to avoid saturation), and modulation amplitudes.

The quantified data in Table S1 were obtained using the corresponding molecular weight. In the case of MORFs, the molecular weight of the unit cell was used.

$$N = n_{s(std)} \frac{n_{s(sample)}}{(N_A m)/M_w}$$

Table S1. Experimental and calculated values from ESR spectra to quantify the number of spin centres per molecule or unit cell.

	**m (mg)**	**M_w (g/mol)**	**Area (DI)**	**n_s**	**N**
**$PTMTC^R$**	4,2	788.69	172335299	2.73E+21	0.85
**MORF-80**	10.2	1338.54	212212849	3.36E+21	0.73
**MORF-60**	9.4	1338.54	148803665	2.36E+21	0.56
**MORF-40**	9.1	1338.54	62131075	9.84E+20	0.24

5. Raman spectroscopy

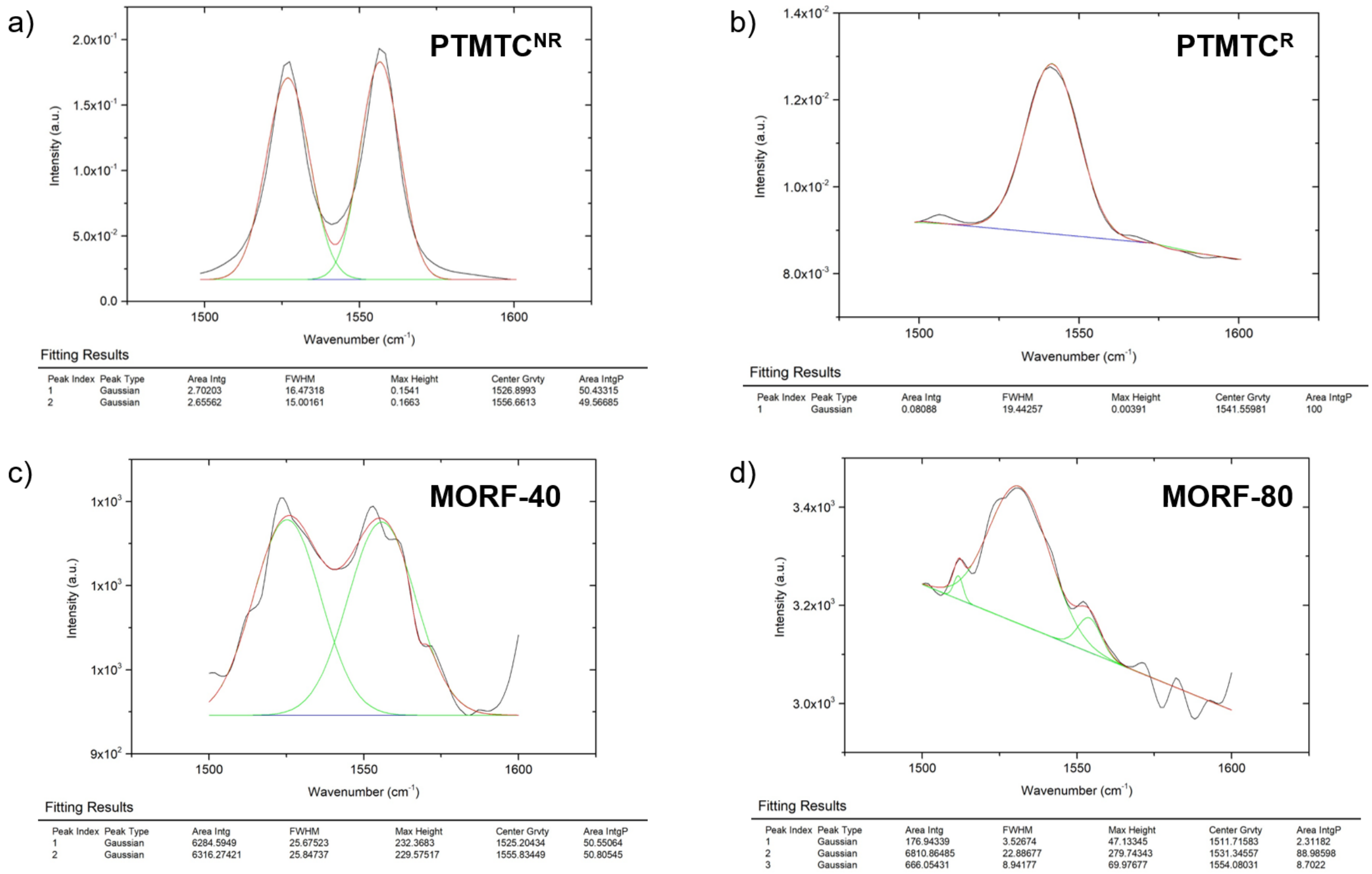

a) Fitting Results

Peak Index	Peak Type	Area Intg	FWHM	Max Height	Center Grvty	Area IntgP
1	Gaussian	2.70203	16.47318	0.1541	1526.8993	50.43315
2	Gaussian	2.65562	15.00161	0.1663	1556.6613	49.56685

b) Fitting Results

Peak Index	Peak Type	Area Intg	FWHM	Max Height	Center Grvty	Area IntgP
1	Gaussian	0.08088	19.44257	0.00391	1541.55981	100

c) Fitting Results

Peak Index	Peak Type	Area Intg	FWHM	Max Height	Center Grvty	Area IntgP
1	Gaussian	6284.5949	25.67523	232.3683	1525.20434	50.55064
2	Gaussian	6316.27421	25.84737	229.57517	1555.83449	50.80545

d) Fitting Results

Peak Index	Peak Type	Area Intg	FWHM	Max Height	Center Grvty	Area IntgP
1	Gaussian	176.94339	3.52674	47.13345	1511.71583	2.31182
2	Gaussian	6810.86485	22.88677	279.74343	1531.34557	88.98598
3	Gaussian	666.05431	8.94177	69.97677	1554.08031	8.7022

Figure S2. Raman spectra of a) $PTMTC^{NR}$, b) $PTMTC^{R}$, c) **MORF-40**, and d) **MORF-80** in the 1500–1600 cm^{-1} region, showing the experimental spectra (black), fitted profiles (red), and deconvoluted individual contributions (green).

6. Kelvin Probe Force Microscopy

KPFM measurements were performed at room temperature in a rarefied atmosphere (air pressure 3×10^{-2} Torr) using Park NX HiVac microscope (Park Systems). A supersharp AFM probe SSS-NCHR10 (Nanosensors, Switzerland) with the tip curvature radius below 2 nm and the resonance frequency of 249.7 kHz has been used. The amplitude of the free vibrations was set at 12 nm and non-contact set point was 80% of the free vibrations (9.6 nm) that corresponds to the tip-sample distance of about 16 nm. KPFM measurements were done in a single-pass mode with AC voltage of amplitude 1.5 V and frequency 17 kHz. No DC voltage bias was applied between the tip and the sample. The measured KPFM signal was analyzed by a built-in lock-in amplifier with time constant 1 ms and sensitivity 0.1 V.

The methodology of the measurements is similar to that performed earlier.[5] 10 scans of the size 50×50 nm and 256×256 points were measured in different places of the mixed PTMTC crystals with different NR/R ratios. For each scan line, forward and backward KPFM signals were collected and analysed jointly to insure the reproducibility of the measurements. However, raw scans contain much amount of noise. Therefore, the following pre-treatment scheme was performed using Gwyddion software. First, high frequency noise of electrical origin was removed from the scans using FFT filtration. The instability of the mechanical system especially prominent at small size scans leads to the mismatch even between consequent scans. Therefore, the cross-correlation analysis between forward and backward scans was applied to reveal the regions with high correlation (correlation score higher than 70% of the maximum). The obtained mask was applied to the FFT filtered scans and the average and median values of the KPFM potential were extracted for each region. Such pre-treatment was applied for all pairs of forward and backward scans measured for each crystal. Then, the results were combined together and plotted as histograms. Such analysis allows separating the reproducible signals only thus related to the effect of the sample.

The sample's work function (φ_s) depends on the work function of the tip (φ_t) and the KPFM amplitude, so V_{KPFM}: $\varphi_s = \varphi_t - eV_{KPFM}$, where e is an elementary charge. Therefore, for the same tip, the measured KPFM potential signal is proportional to the sample's work function: $V_{KPFM} = (\varphi_t - \varphi_s)/e$.

7. Fluorescence Microscopy

Confocal fluorescence microscopy measurements were performed using an Andor Dragonfly high speed confocal microscope (Oxford Instruments) equipped with a 465 nm excitation source. Emission spectra were collected from individual crystals at room temperature.

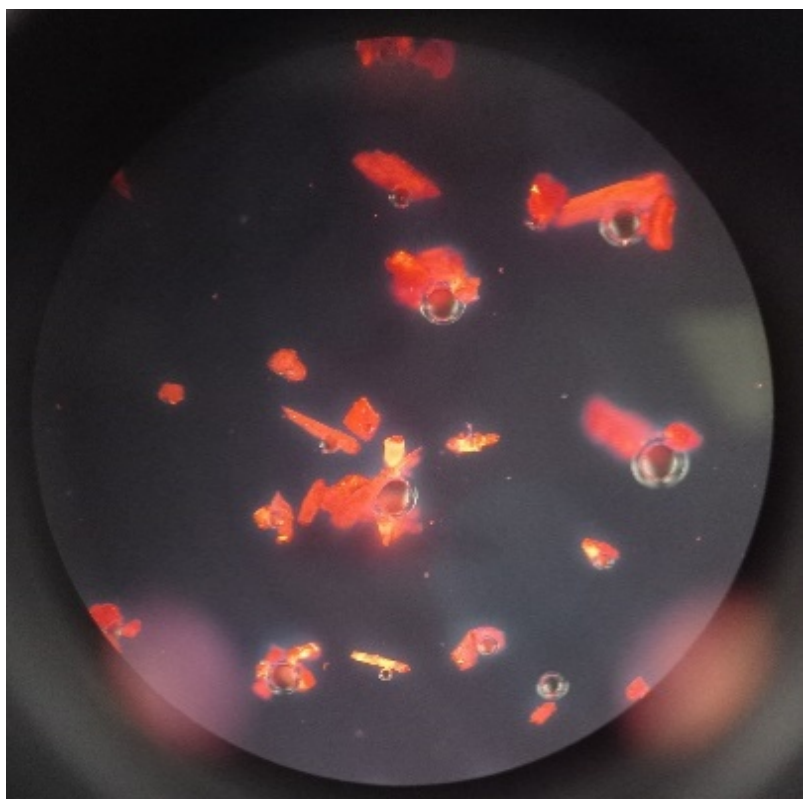

Figure S3. Fluorescence microscope image of **MORF-40** crystals as a representative example. Crystals were embedded in glycerol and observed with a hyperspectral microscope using the UV-DAPI filter.

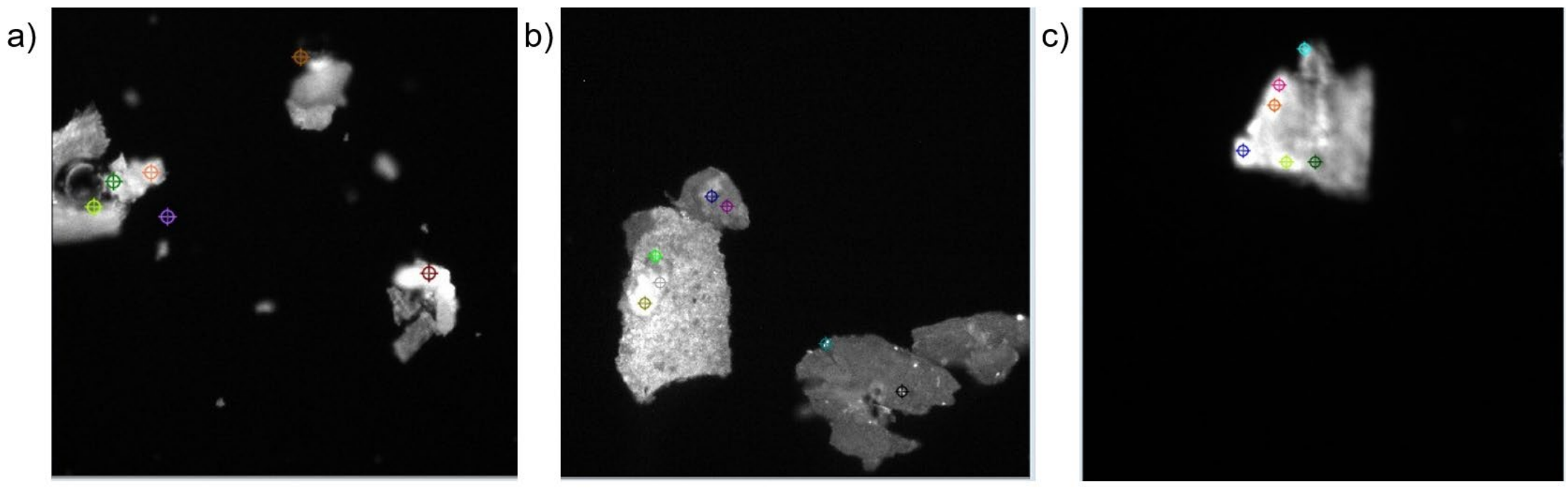

Figure S4. Microscope images of a) **MORF-40**, b) **MORF-60**, and c) **MORF-80** during hyperspectral analysis.

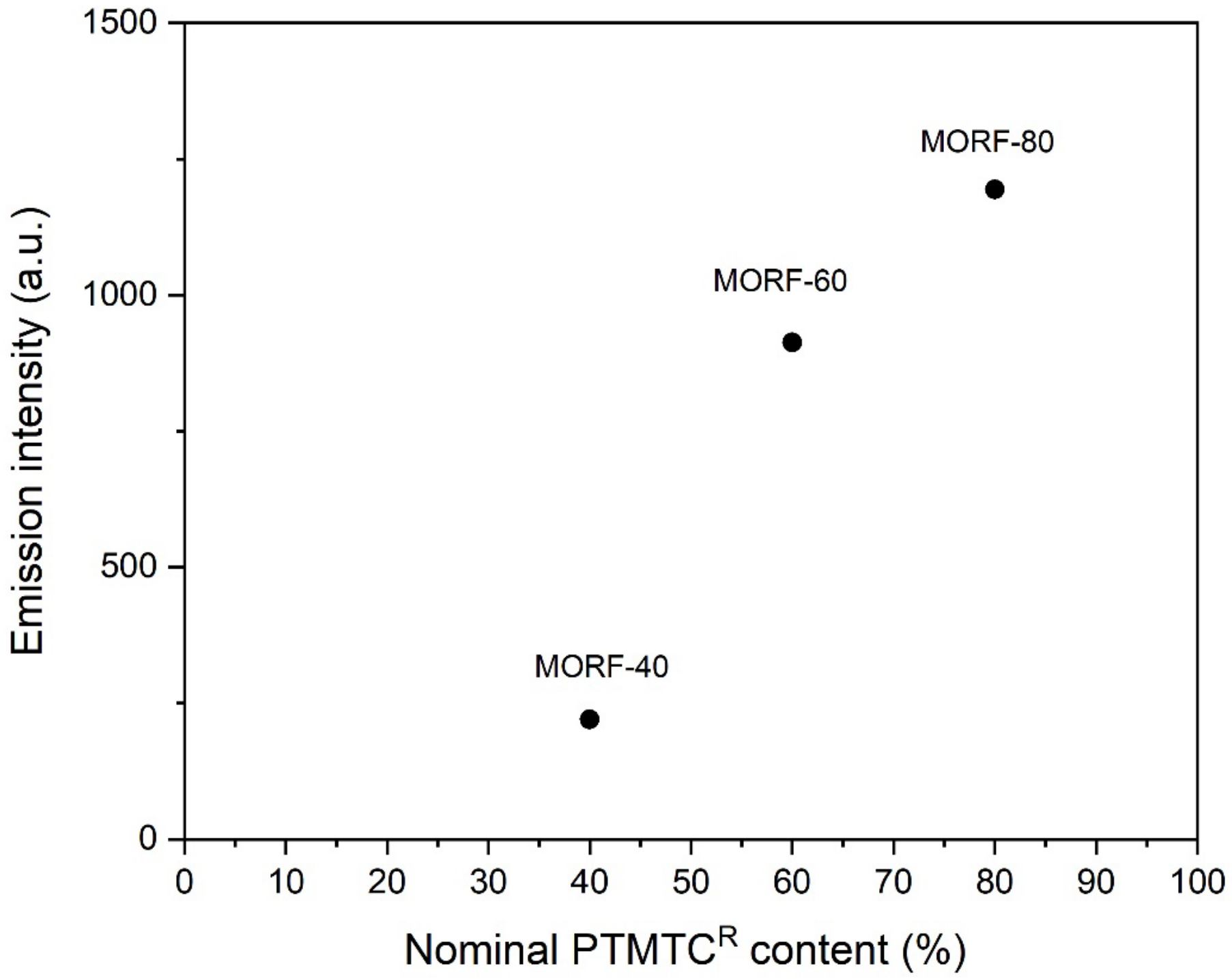

Figure S5. Emission intensity measured at 605 nm as a function of the nominal $PTMTC^R$ radical content for **MORF-40**, **MORF-60**, and **MORF-80**, obtained from confocal fluorescence microscopy under identical acquisition conditions.

8. References

1. T. Kottke and D. Stalke, *J. Appl. Crystallogr.* **1993**, *26*, 615–619.
2. Y. Oxford Diffraction Ltd, England, in Oxford Diffraction, **2022**, CrysAlis PRO, Rigaku V1.171.142.173a.
3. G. M. Sheldrick, *Acta Crystallogr. Sect. A*, **2015**, *71*, 3–8.
4. C. B. Hübschle, G. M. Sheldrick and B. Dittric, *J. Appl. Crystallogr.*, **2011**, *44*, 1281– 1284.
5. G. Valente, P. Ferreira, M. A. Hernández-Rodríguez, C. D. S. Brites, J. Amaral, P. Zelenovskii, F. A. A. Paz, S. Guieu, J. Rocha, M. Souto. *Chem. Mater.* **2024**, *26*, 1333-1341.
